# Glutathione/Selenite Interaction Products as Potent Vascular Smooth Muscle Tone Modulators with Antihypertensive Effects in Spontaneously Hypertensive Rats

**DOI:** 10.1007/s12011-026-05188-1

**Published:** 2026-06-24

**Authors:** Lenka Tomasova, Peter Balis, Marian Grman, Anna Zemancikova, Sona Cacanyiova, Miroslav Chovanec, Karol Ondrias, Anton Misak

**Affiliations:** 1 Institute of Clinical and Translational Research, Biomedical Research Center, Slovak Academy of Sciences, Dubravska cesta 9, Bratislava, 84505 Slovak Republic; 2 Institute of Normal and Pathological Physiology, Centre of Experimental Medicine, Slovak Academy of Sciences, Sienkiewiczova 1, Bratislava, 81371 Slovak Republic; 3 Cancer Research Institute, Biomedical Research Center, Slovak Academy of Sciences, Dubravska cesta 9, Bratislava, 84505 Slovak Republic

**Keywords:** Glutathione, Selenite, Reactive selenium species, Spontaneous hypertensive rat, Vasoactive effects, Hemodynamic parameters

## Abstract

Selenium is an essential trace element important for human health and its deficiency is typically compensated through dietary supplementation, including selenite (SeO_3_^2−^). In biological systems, SeO_3_^2−^ undergoes multistep reduction, generating selenium species with distinct oxidation states and redox properties. In this study, we investigated the cardiovascular effects of a glutathione/selenite mixture (GSH/SeO_3_^2−^) in spontaneously hypertensive rats (SHRs). Arterial pulse waves were recorded using a pressure microcatheter and vascular tension in isolated arteries was assessed by isometric wire myography. Intravenous administration of GSH/SeO_3_^2−^ induced a pronounced hypotensive response, accompanied by reductions in heart rate and augmentation index. Furthermore, GSH/SeO_3_^2−^ elicited heterogeneous vasomotor responses across mesenteric, femoral, and aortic vascular beds. Marked vasorelaxation observed in the mesenteric artery, a representative resistance vessel, appeared to be associated with the formation of elemental selenium (Se^0^) and was partially endothelium-independent. In contrast, aortic segments exhibited marked vasoconstriction. Notably, co-application of superoxide dismutase and catalase attenuated this response, suggesting that aortic segments are more sensitive to ROS generated during the GSH/SeO_3_^2−^ interaction. The hypotensive effect of GSH/SeO_3_^2−^ was preserved in SHRs pretreated with losartan and captopril, indicating additional antihypertensive efficacy beyond the pharmacological inhibition of renin-angiotensin-aldosterone system. Importantly, the hypotensive action of GSH/SeO_3_^2−^ was dependent on sympathetic nervous system activity.

**Supplementary Information:**

The online version contains supplementary material available at 10.1007/s12011-026-05188-1.

## Introduction

Selenium (Se) is an essential trace element that plays a crucial role in human health. Depending on the level of daily intake, both beneficial and adverse effects of Se consumption have been well documented [1–3]. Se deficiency has been associated with several cardiovascular disorders, including myocardial infarction and heart failure, highlighting its importance in the maintenance of cardiovascular homeostasis [4–6]. Moreover, serum Se concentrations exhibit a U-shaped relationship with systolic blood pressure and its deficiency is associated with an increased risk of hypertension [7–9].

The biological function of Se is primarily attributed to its role as a redox-active component of selenoproteins, which are key elements of cellular antioxidant defense systems. In cases of Se deficiency, inorganic sodium selenite (Na_2_SeO_3_) and organic selenomethionine (SeMet) are commonly used as dietary supplements. The metabolism of selenite (hereinafter referred to as SeO_3_^2−^, including its protonated forms) involves its reduction by reduced glutathione (GSH) in the intestinal lumen and/or bloodstream, followed by hepatic uptake and conversion to selenophosphate, which is subsequently utilized for the synthesis of selenoamino acids in peripheral tissues [10]. Several studies have reported the chemical interaction between GSH and SeO_3_^2−^ (GSH/SeO_3_^2−^), characterizing reaction products, such as selenodiglutathione (GSSeSG), selenopersulfides (RSSe^−^), and selenide (Se^2–^), indicating that Se(IV) reduction proceeds through a multistep process [11–14]. This redox chemistry is accompanied by the generation of reactive oxygen species (ROS), including superoxide anion radical (^●^O_2_^−^) and hydrogen peroxide (H_2_O_2_) [15, 16]. Under specific conditions, SeO_3_^2−^ reduction may also yield elemental selenium (Se^0^), which can self-assemble into Se nanoparticles. Notably, Se nanoparticles have been reported to exhibit enhanced antioxidant activity compared with bulk Se forms while demonstrating reduced toxicity [17]. Collectivelly, these findings underscore the complex redox chemistry of Se, which gives rise to reactive species with distinct biological activities that may extend beyond its canonical selenoprotein-mediated functions.

In our previous work, we demonstrated that products formed during the interaction of GSH with SeO_3_^2−^ possess reducing properties, cleave plasmid DNA (pDNA), and lower blood pressure (BP) and vascular tone in mesenteric arteries in normotensive rats [18]. The present study aimed to extend these findings by evaluating the cardiovascular effects and underlying pharmacological mechanisms of GSH/SeO_3_^2−^ in spontaneously hypertensive rats (SHRs). In addition, we evaluated the antihypertensive efficacy of GSH/SeO_3_^2−^ in SHRs pretreated with losartan (LOS) and captopril (CAP), two standard-of-care antihypertensive agents targeting the renin-angiotensin-aldosterone system (RAAS). Given that arterial hypertension remains the leading modifiable risk factor for cardiovascular morbidity and mortality worldwide [19], our findings may contribute to the identification of novel antihypertensive strategies based on Se-containing compounds.

## Materials and Methods

### Chemicals and Solutions

The following chemicals were purchased from Sigma-Aldrich (Schnelldorf, Germany): sodium phosphate monobasic (NaH_2_PO_4_; S5011), sodium phosphate dibasic (Na_2_HPO_4_; S7907), sodium selenite (Na_2_SeO_3_; 214485), diethylenetriaminepentaacetic acid (DTPA; D6518), L-glutathione reduced (GSH; G4251), losartan potassium (LOS; 61188), angiotensin II human (AngII; A9525), captopril (CAP; C4042), serotonin creatinine sulfate monohydrate (Ser; H7752), glibenclamide (G0639), norepinephrine (NOR; A7257), Nω-Nitro-L-arginine methyl ester hydrochloride (L-NAME; N5751), pentolinium tartrate (pentolinium; R278084), tetraethylammonium chloride (TEA; 86614), superoxide dismutase (SOD; S7446-15KU), 2-(4-carboxyphenyl)−4,5-dihydro-4,4,5,5-tetramethyl-1 H-imidazol-1-yloxy-3-oxide potassium salt (^●^cPTIO; C221), catalase (CAT; C9322), dimethyl sulfoxide (DMSO; D4540), potassium dioxide (KO_2_; 278904) and hydrogen peroxide (H_2_O_2_; 85321). Isoflurin (100% Isoflurane inhalation vapour) was purchased from Vetpharma (Barcelona, Spain). Physiological saline (0.9% NaCl) was bought from B. Braun (Melsungen, Germany).

Stock solutions listed below were prepared freshly in 0.9% NaCl: AngII, LOS, CAP and SeO_3_^2−^. The following stock solutions were prepared using ultrapure deionized water (ddH_2_O): ^●^cPTIO, TEA, L-NAME, H_2_O_2_, Ser, NOR or organic solvent DMSO: glibenclamide and KO_2_. Both enzymes SOD and CAT were prepared in 50 mM Sodium Phosphate (pH 7.2). For subsequent use in the in vivo experiments, all stock solutions were diluted in physiological saline.

A stock solution of GSH was prepared in 100 mM sodium phoshate, 100 µM DTPA, 7.4 pH buffer. A ultrapure ddH_2_O (conductivity ≤ 54.0 nS/cm) was used to prepare stock solutions.

### Measurement of Arterial Pulse Wave (APW) Parameters (APW-Ps) and their Presentation

Adult male SHRs (*n* = 32; 280–320 g; 17–19 weeks old) were purchased from the Department of Toxicology and Laboratory Animals Breeding, Centre of Experimental Medicine, Dobra Voda and housed at the Central Animal House Facility of Pavilion of Medical Sciences (registration number SKUCH 04022, Bratislava, Slovak Republic). The rats were maintained under standard conditions at a temperature of 22–24 °C on a 12:12 h dark–light cycle (lights on from 06.00 h to 18.00 h) and provided a standard pellet diet and tap water *ad libitum*. Isoflurane (ISO) was used as the inhalational anesthetic. The rats were initially placed in an induction chamber flushed with 5 vol % ISO in 100% oxygen until the loss of the righting reflex. The rats were then placed on a heated pad (37 °C) and further anesthetized with 3 vol % ISO in 100% oxygen (0.9 L/min) administered using a nose cone. To prepare the GSH/SeO_3_^2−^ mixture, 150 µL of 200 mM GSH in phosphate buffer (100 mM sodium phosphate, 100 µM DTPA, pH 7.4) were mixed with 50 µL of 100 mM SeO_3_^2−^ in 0.9% NaCl (final pH ~ 4) and incubated in a microtube for 45 s at 23 ± 1 °C. After incubation, the GSH/SeO_3_^2−^ mixture (150/25 mM) was administered intravenously (i.v.) into the cannulated right jugular vein at a bolus dose of 500 µL/kg over 15 s. The calculated dose of the GSH/SeO_3_^2−^ mixture was 75/12.5 µmol/kg. The concentrations of GSH and SeO_3_^2−^ were selected to ensure robust redox interactions and to yield measurable biological responses. An angiotensin-converting enzyme (ACE) inhibitor CAP (12.5 µmol/kg), angiotensin receptor (ATR) blocker LOS (12.5 µmol/kg), GSH (75 µmol/kg) or SeO_3_^2−^ (12.5 µmol/kg) were administered into the right jugular vein in the same bolus dose of 500 µL/kg over 15 s. An exogenous angiotensin II (AngII; 0.2 nmol/kg) was used to confirm ATRs blockade by LOS. The involvement of NO-mediated pathways was tested by NO synthase inhibition with L-NAME (25 mg/kg). The contribution of sympathetic nervous sytem (SNS) in blood pressure management was examined by administration of ganglion blocker pentolinium (5 mg/kg). In the same way, saline (0.9% NaCl) and vehicle (150 µL of 100 mM phosphate buffer mixed with 50 µL of saline, pH adjusted to ~ 4) were administered. The left common carotid artery (*a. carotis communis*) was cannulated for the insertion of a FISO LS 2 F microcatheter pressure transducer (Harvard Apparatus, Holliston, MA, USA) to record APWs. The principle of APWs measurement and APW-Ps definitions are detailed in our previous studies [20, 21]. To characterize the time-dependent cardiovascular response to treatment, six APW-Ps were derived from APWs: systolic BP (SysBP), diastolic BP (DiaBP), pulse BP (PulBP), augmentation index relative (AIr), heart rate (HR) and arterial d*P*/d*t*_max_.

**Fig. 1:**
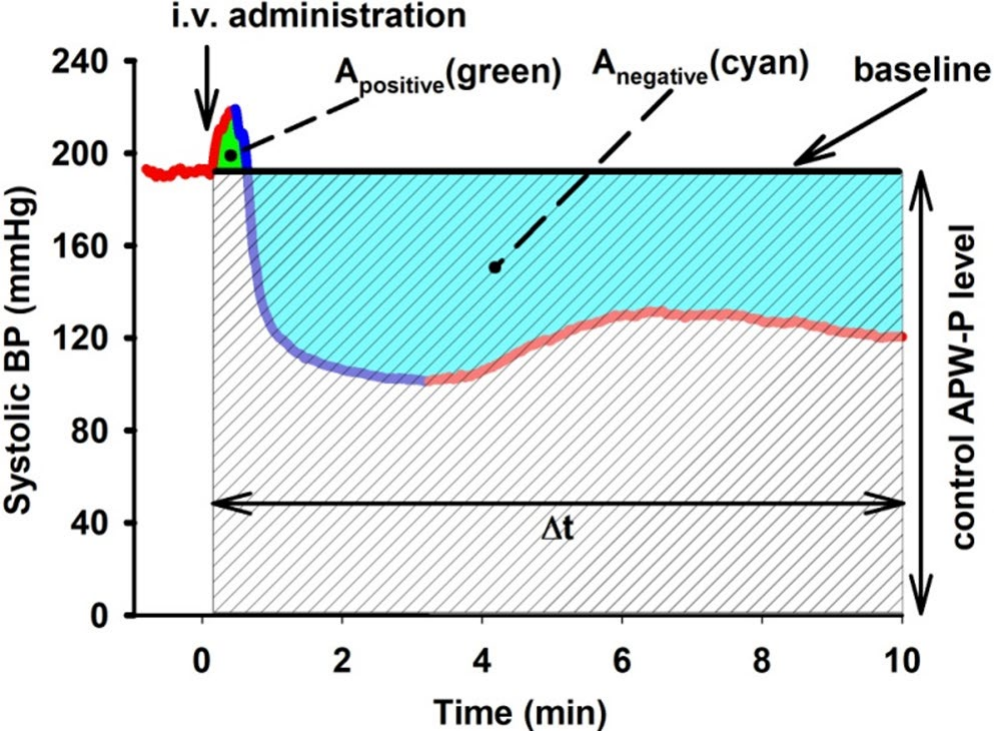
Explanatory diagram illustrating the principles of APW-P presentation. In this example, the APW-P, represented by systolic BP, crosses the baseline and creates short positive (green) and pronounced negative (cyan) segments corresponding to A_positive_ and A_negative_ areas, respectively. The APW-P baseline is defined as a 30-s mean APW-P recorded before intavenous (i.v.) administration and represents the control APW-P level. The total area of the positive/negative components is compared to the basal area (hatched region), which is calculated as the product of the baseline and interval ∆*t*. The blue trace represents the descending segment of SysBP and was used for correlation with the corresponding segments of five additional parameters

Since each record produced a different APW-P profile in time, often characterized with a multi-phasic behavior, we calculated the areas of positive and negative components formed above and below APW-P baseline (Fig. 1).The total positive or negative area was then normalized to the basal area using the following formula:

positive change of APW-P area (%)

$$\\\begin{array}{c}positive\;change\;of\;APW-P\;area\;(\%)\\\:=\:\frac{\sum\:A_\mathrm{positive}}{\:\mathrm{basal}\:\mathrm{area}}\:\mathrm{x}\:100\mathrm{\%}\end{array}$$

$$\\\begin{array}{c}\:negative\;change\;of\;APW-P\;area\;(\%)\\=\:\frac{\sum\:A_\mathrm{negative}}{\:\mathrm{basal}\:\mathrm{area}}\:\mathrm{x}\:100\mathrm{\%}\end{array}$$

where *A*_positive_ and *A*_negative_ represent the areas under the curve of positive and negative components, respectively. The basal area was calculated as the baseline value multiplied by the time interval ∆*t*. Theoretically, this quantity represents the integrated baseline signal of a given APW-P over the interval ∆*t*, which typically lasted 10 min.

A consistent feature observed following the administration of GSH/SeO_3_^2−^ across all experiments was a pronounced decrease in SysBP, followed by a transient rise and eventual stabilization (a representative trace is shown in Fig. 1). Given the high reproducibility of this response pattern, the descending phase of the curve (blue segment in Fig. 1) was used for correlation analyses between SysBP and the corresponding values of HR, DiaBP, PulBP, AIr and d*P*/d*t*_max_.

### Measurement of the Vasoactive Effect of GSH and SeO_3_^2−^ Interaction Products

Adult male SHRs (*n* = 6, weight 303 ± 8.6 g; 17–19 weeks old) were obtained and housed as described above. After brief carbon dioxide anesthesia, the rats were euthanized by decapitation and vascular rings were isolated from the mesenteric artery (*a. mesenterica*), femoral artery (*a. femoralis*) and thoracic aorta. Mesenteric arteries were isolated from Wistar rats (*n* = 3, weight 336 ± 39 g; 18 weeks old) under the same conditions as described for SHRs. Functional studies of the isolated arterial rings were performed as described in our previous study [22], with some modifications.

Approximately 1.6 mm segments of small mesenteric arteries (first-order branches of the inferior mesenteric artery), as well as femoral arteries, and thoracic aorta were isolated and mounted as ring preparations in a Mulvany–Halpern wire myograph (Dual Wire Myograph System 410 A, DMT A/S, Aarhus, Denmark). Vascular reactivity was measured isometrically in modified physiological salt solution (PSS; in mM: NaCl 118.99, KCl 4.69, NaHCO₃ 25, MgSO₄ 1.17, KH₂PO₄ 1.18, CaCl₂ 2.5, Na₂EDTA 0.03, glucose 5.5; pH 7.4). Mounted vessels were maintained in continuously oxygenated PSS (95% O₂ and 5% CO₂) at 37 °C. The segments of mesenteric artery were preconstricted using NOR (1 µM), femoral artery with Ser (1 µM) and thoracic aorta with NOR (10 µM). Once a stable contractile tone was achieved, GSH was applied to the organ bath (5 or 10 mL volume chambers), followed by SeO_3_^2–^ (~ 5 s later) to attain the final bath concentrations of 500 and 40 µM. These concentrations corresponded to the effective molar ratio used in our previous study [18]. The notation GSH+SeO_3_^2−^ was used to distinguish the sequential application of GSH and SeO_3_^2−^ in the functional studies on isolated arteries from in vivo experiments, where both compounds were i.v*.* applied as the GSH/SeO_3_^2−^ mixture. To investigate the mechanisms underlying vasomotor responses, pharmacological profiling was conducted using NO synthase inhibition with L-NAME (300 µM), blockade of K_ATP_ channel with glibenclamide (40 µM), and inhibition of large-conductance Ca²⁺-activated K⁺ channels (BK_Ca_) with TEA (1 mM). To investigate the role of the endothelium in vasoactive effects of GSH+SeO_3_^2−^, the endothelium was mechanically removed. Endothelial removal was verified by the complete abolition of acetylcholine-induced relaxation in preconstricted vessels. Vascular reactivity was assessed within 12 min after the application of GSH+SeO_3_^2−^. Because the contractile response exhibited a complex and multiphasic profile, changes in vascular tone were quantified using a series of indicative points along the tension curve. These included distinct inflection points as well as the endpoint at the 12th min. The effect of GSH+SeO_3_^2−^ on vascular tension was expressed as the relative change in tension (%) of the preconstricted artery at the indicative points (∆*T*/*T*_0_ × 100%, where ∆*T* represents the difference between the baseline tension *T*_0_ and the actual tension *T* at the indicative point). This standardization allowed comparison of responses across vessels and experimental groups despite variability in basal tone.

### UV–Vis Spectra of GSH/SeO_3_^2−^ and Reduction of the ^●^cPTIO Radical

UV-Vis spectrophotometry was employed to characterize the chemical properties of the GSH/SeO_3_^2−^ mixtures used in this study, considering various factors such as reaction time, GSH: SeO_3_^2−^molar ratio, and the presence of SOD and CAT. UV–Vis absorption spectra (optical path length 10 mm) were recorded using a Shimadzu 1800 spectrophotometer (Kyoto, Japan) equipped with a Peltier temperature-control system. Detailed experimental parameters are provided in the legends accompanying the relevant figures.

To evaluate the efficiency of GSH/SeO_3_^2−^ in reducing the ^●^cPTIO radical, an aliquot of the GSH/SeO_3_^2−^ mixture was added to 100 mM sodium phosphate buffer (100 µM DTPA, pH 7.4) containing 100 µM ^●^cPTIO. The reduction of ^●^cPTIO by GSH/SeO_3_^2−^ was monitored by measuring the decrease in absorbance (ABS) at 560 nm [18].

### pDNA Cleavage Assay

A pDNA cleavage assay using the pBR322 plasmid (New England BioLabs, 134 Inc., N3033 L, Ipswich, MA, USA) was performed as previously published [23]. All samples contained 0.2 µg of pDNA in a final volume of 20 µL phosphate buffer (25 mM sodium phosphate, 50 µM DTPA, pH 7.4). The stock solutions of GSH (200 mM prepared in 100 mM sodium phosphate, 100 µM DTPA, pH 7.4) and SeO_3_^2−^ (100 mM prepared in 0.9% saline) were mixed in a 3:1 ratio to achieve the final concentrations of 150 and 25 mM respectively, and then incubated for 10, 45or 300 s at 24 ± 1 °C. After incubation, 1 µL aliquot was added to 19 µL of the pDNA solution and further incubated for 60 s or 30 min at 37 ± 1 °C. Control samples containing GSH or SeO_3_^2−^ were prepared in parallel, with GSH (200 mM) diluted 3:1 with saline and SeO_3_^2−^ (100 mM) diluted 1:3 with 100 mM sodium phosphate. 1 µL was then added to the pDNA solution. After incubation, the reaction mixtures were subjected to 0.6% agarose gel electrophoresis. The integrated densities of the two pBR322 forms (supercoiled and nicked circular) in each lane were quantified using Image Studio analysis software (LI-COR Biotechnology, Bad Homburg, Germany) to determine the efficiency of pDNA cleavage.

### Data Analysis

Data analysis and visualization were performed using SigmaPlot 12.5 (Systat Software, San Jose, CA, USA) or GraphPad Prism 9 (GraphPad, La Jolla, CA, USA). Unless otherwise indicated, data are presented as means ± standard deviation (SD), with *n* denoting the number of independent experiments. The normality of the data distribution was assessed using the Shapiro-Wilk normality test and Q-Q plots. A one-way ANOVA with Tukey’s post hoc test or two-way ANOVA followed by Sidak’s multiple comparison test was used for between-group comparisons. *P* value < 0.05 was considered statistically significant.

## Results

### The Effect of GSH, SeO_3_^2−^ and GSH/SeO_3_^2−^ on APW-Ps of SHRs

**Fig. 2:**
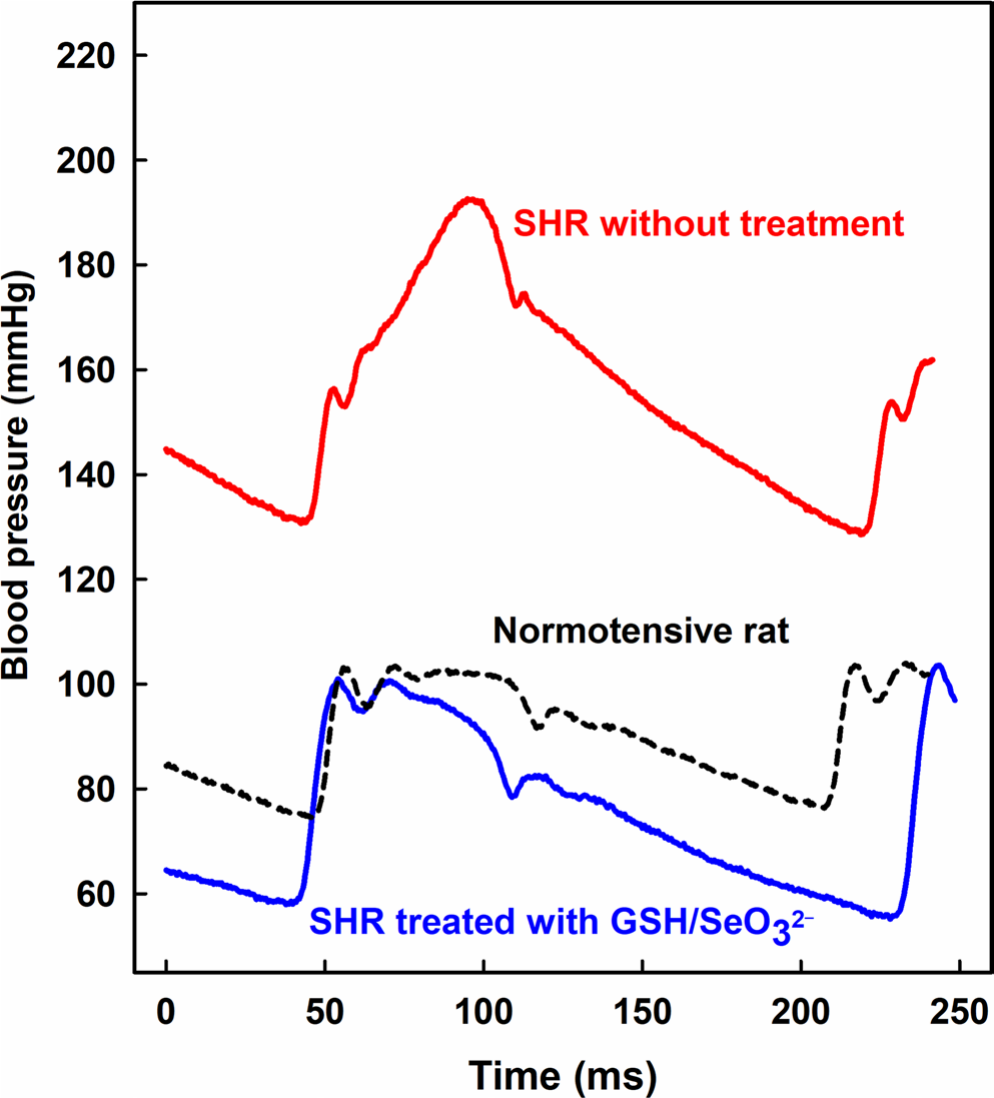
Representative APW observed during blood pressure measurement in left common carotid artery of an anesthetized SHR. Basal APW before (red) and after (blue) i.v. administration of the GSH/SeO_3_^2−^ mixture incubated for 45 s at 23 ± 1 °C. Six APW-Ps were derived from APWs, and their representative time courses are presented graphically in Fig. 3. For comparison, the APW recorded in a normotensive male rat is shown (black dashed line; data published in [25])

**Fig. 3:**
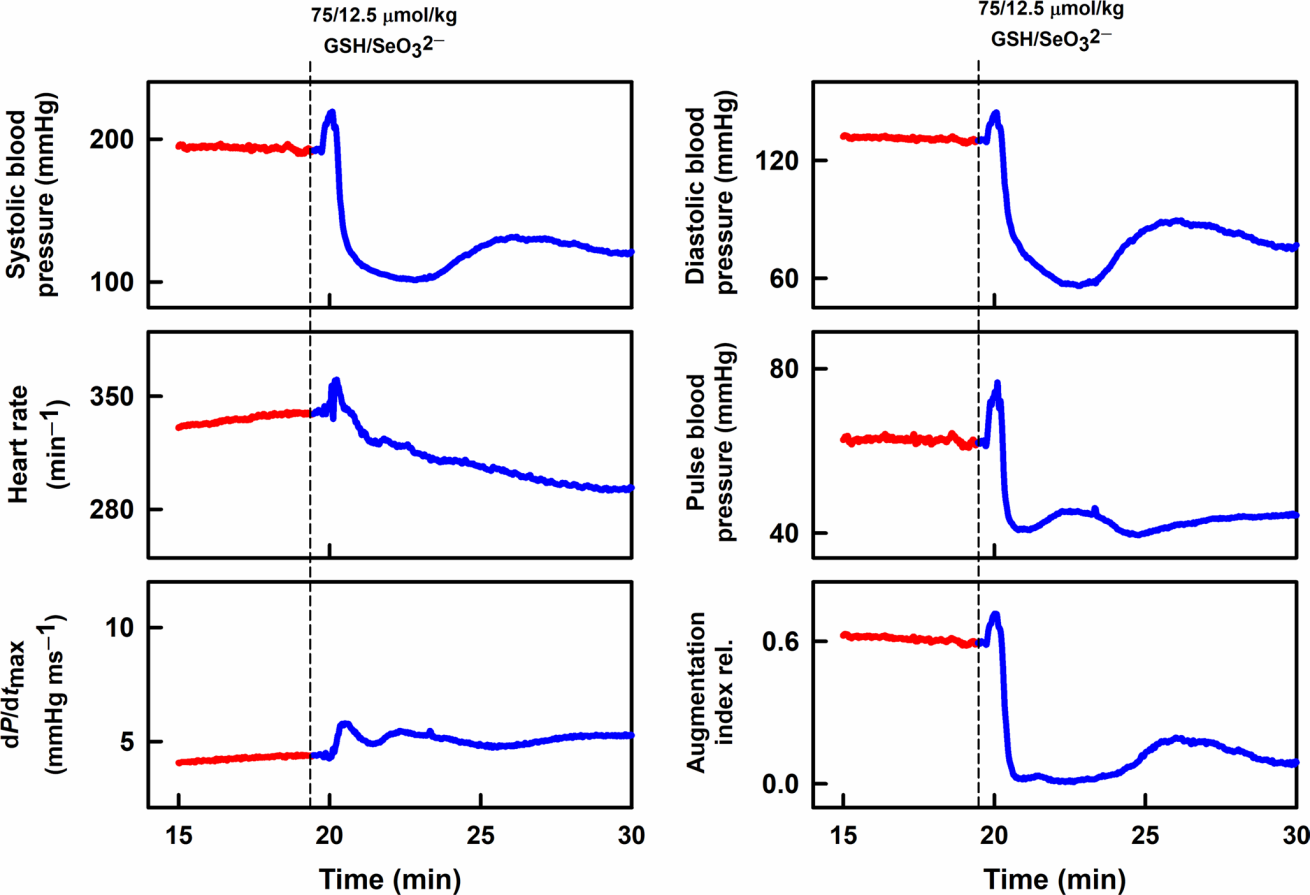
Representative time-resolved changes in APW-Ps acquired in an anesthetized SHR before (red) and after (blue) i.v. administration of GSH/SeO_3_^2−^. The bolus dose of GSH/SeO_3_^2−^ (150/25 mM) was standardized based on the body weight as 75/12.5 µmol/kg. Vertical black dashed lines indicate the onset of each administration with a duration of 15 s

The stock solution of GSH/SeO_3_^2−^ (150/25 mM) turned from colorless to faint yellow−green approximately 40–45 s after mixing (Fig. S1), then progressed to orange and finally crimson after a 30-min incubation at 23 ± 1 °C. The orange − red color indicated the formation of elemental Se (Se^0^) aggregates [24], which began to accumulate at the bottom of the glass vial. Therefore, we used an incubation time of 45 s for i.v. administration, at which the chemical reaction(s) between GSH and SeO_3_^2−^ had reached a certain stage, with distinct intermediate products already formed. It should also be noted that the bolus administration lasted 15 s. The basal APWs of SHRs obtained in the control conditions exhibited a typical ‘high BP’ profile (Fig. 2). The mean basal systolic and diastolic BP of 32 SHRs were 183.3 ± 19.6 and 124.9 ± 12.5 mmHg, respectively. After i.v. administration of GSH/SeO_3_^2−^ (bolus dose of 75/12.5 µmol/kg), the APW underwent significant changes, resembling that observed in normotensive rats. Consequently, all APW-Ps derived from the APWs were affected (Fig. 3). It was shown that the pharmacological effect of GSH/SeO_3_^2−^ may persist for more than 20 min (Figs. S2 and S3).

After administration of GSH/SeO_3_^2−^, systolic and diastolic BP reached minimal values of 113.1 ± 17.6 and 67.6 ± 18.3 mmHg, respectively. By the 10th minute, the antihypertensive effect of the mixture remained still significant, with systolic and diastolic BP being 140.4 ± 17.9 and 91.6 ± 13.3 mmHg, respectively. However, the progression of APW-Ps was not linear, as most experiments exhibited multiphasic alterations during the 10-min analysis period (Fig. S4). Nearly all parameters typically began with a short positive phase, which was immediately followed by an opposite change. In contrast, d*P*/d*t*_max_ generally started with a short negative phase. Similar alterations, however with different phase duration, were also demonstrated when SeO_3_^2−^ (Fig. S5) and GSH (Fig. S6) were applied separately.

**Fig. 4:**
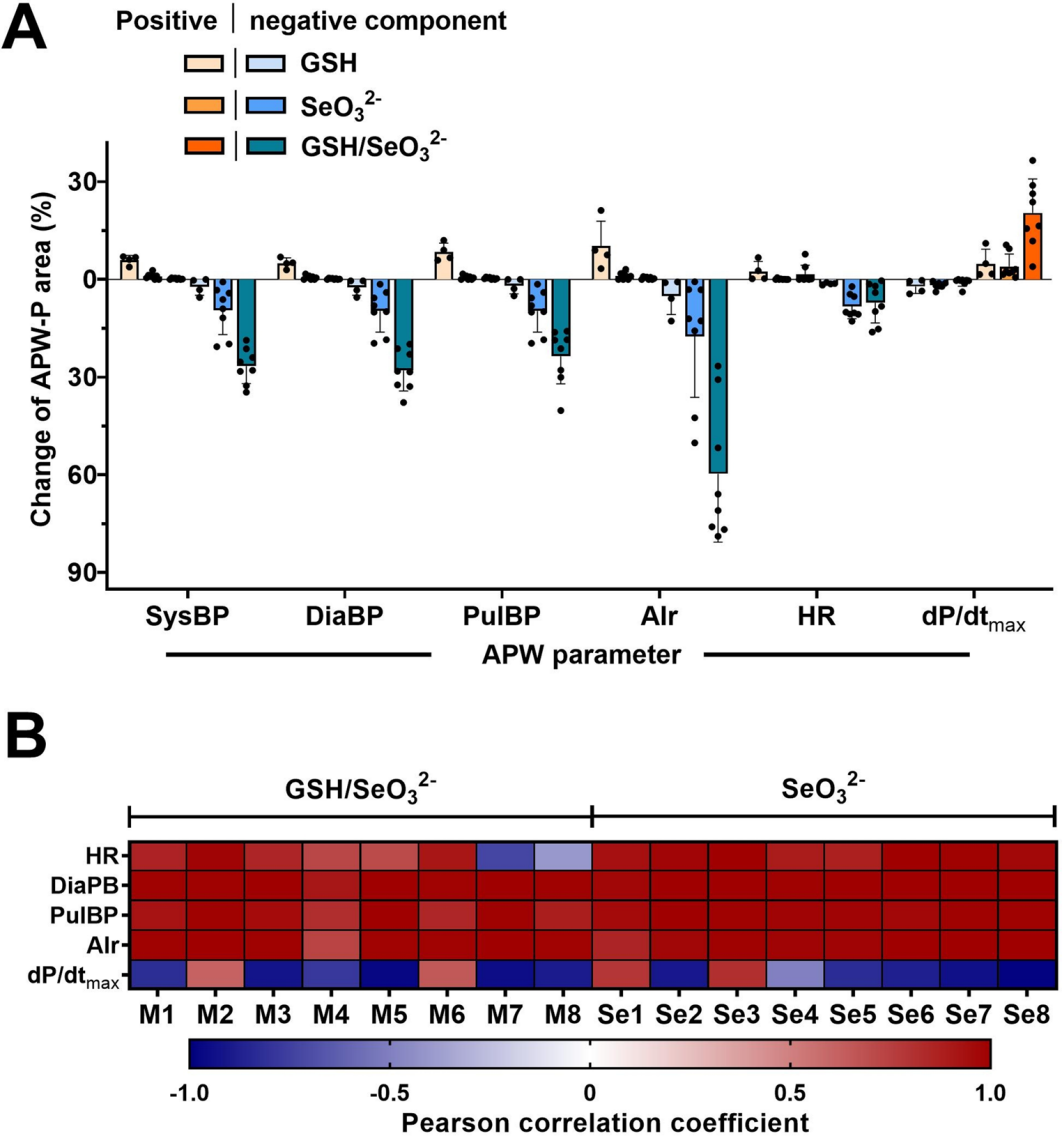
The effect of GSH, SeO_3_^2−^ and GSH/SeO_3_^2−^ on APW-Ps. **(A)** After i.v. administration of GSH (75 µmol/kg), SeO_3_^2−^ (12.5 µmol/kg) or GSH/SeO_3_^2−^ (75/12.5 µmol/kg), the positive/negative components in APW-Ps changes were analyzed during the first 10 min (see Fig. 1 for clarification). The total increase (orange-shaded bars) and decrease (blue-shaded bars) in APW-Ps were evaluated by calculating the area under the curve and expressed as a proportion of the basal APW-P area. Data are presented as individual values with means ± SD and originate from Figs. S4 (GSH/SeO_3_^2−^, *n* = 8), S5 (SeO_3_^2−^, *n* = 8) and S6 (GSH, *n* = 4). **(B)** Heat map of Pearson correlation coefficients between the descending phase of SysBP and corresponding DiaBP, PulBP, AIr, HR, and d*P*/d*t*_max_ following administration of the GSH/SeO_3_^2−^ mixture (8 experiments M1-M8) and SeO_3_^2−^ (8 experiments Se1-Se8). The degree of correlation is indicated by blue (negative) and red (positive), as shown in the color scale. All correlations were statistically significant (*P* < 0.0001)

The area-under-curve analysis revealed that the positive and negative components above and below the APW-P baseline have a distinct pattern for each treatment (Fig. 4). Notably, the most pronounced pressor effect associated with initial increase in BP had GSH, while it was less marked for SeO_3_^2−^ and GSH/SeO_3_^2−^. However, the administration of saline and vehicle elicited a short pressor phase, although the BP increase was minimal with the saline and bit more pronounced with the vehicle (Fig. S7). The results indicate that (i) an immediate pressor reaction to bolus administration was not consistently present, and (ii) the vehicle exerted a more pronounced effect than saline. Thus, the initial pressor phase likely represents a systemic reaction to bolus administration itself, which is quickly counterbalanced by a strong pharmacological effect of SeO_3_^2−^ and GSH/SeO_3_^2−^, leading into an opposite phase. Because the contribution of the administration of saline (used as the SeO_3_^2−^ preparation medium) or vehicle (used as the GSH preparation medium) cannot be omited in ISO-anesthetized SHRs, we focused our analysis on the negative (positive for d*P*/d*t*_max_) components of GSH/SeO_3_^2−^ and SeO_3_^2−^ only, which can be interpreted with confidence as reflecting pharmacological effects.

**Fig. 5:**
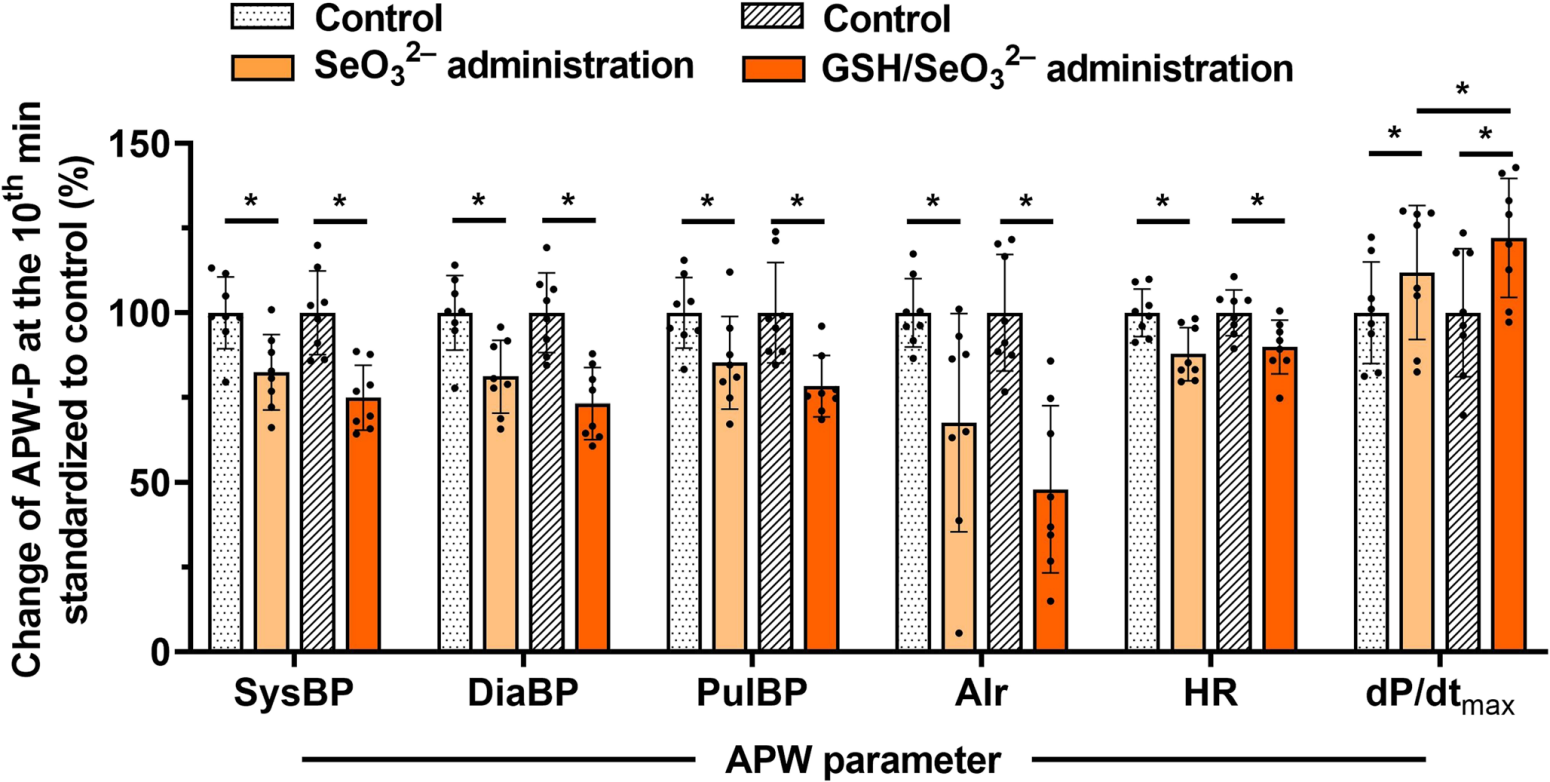
Relative change in APW-Ps at the 10th min after SeO_3_^2−^ or GSH/SeO_3_^2−^ administration. Each APW-P was evaluated at the 10th minute and expressed as a percentage of the mean basal APW-P, which was defined as 100%. The basal APW-P (control, 0th minute) was calculated as the 30-s mean APW-P recorded prior to administration. Data are presented as individual values with means ± SD and originate from Figs. S4 (GSH/SeO_3_^2−^, *n* = 8) and S5 (SeO_3_^2−^, *n* = 8). Statistical analysis was performed using a two-way ANOVA followed by Šidák’s multiple comparison test, with *P < 0.05 (statistical results are provided in Tab. S1)

Qualitative analysis indicated a decrease in SysBP, DiaBP, PulBP, AIr and HR, as well as an increase in d*P*/d*t*_max_ (Fig. 4A) after i.v. administration of SeO_3_^2−^ or GSH/SeO_3_^2−^. This observation was supported by correlations between the descending phase of SysBP and 5 additional parameters. Positive correlations were identified for DiaBP, PulBP, AIr, and HR, whereas a negative correlation was observed for d*P*/d*t*_max_ (Fig. 4B). Moreover, the mixture had a more rapid effect on APW-Ps compared to SeO_3_^2−^ alone (Fig. S4 vs. S5), likely due to the delayed “activation” of SeO_3_^2−^ in the reducing environment of bloodstream. Consequently, the effect of SeO_3_^2−^ developed continuously during 10 min, while the mixture caused an immediate and sharp change in APW-Ps. After 10 min, the mixture decreased SysBP and DiaBP slightly more than SeO_3_^2−^ alone, with differences of 7.5 and 8.1%, respectively. Although both treatments kept BP reduced, no statistically significant difference was observed between them at 10th min (Fig. 5).

When GSH/SeO_3_^2−^ (75/12.5 µmol/kg) was administered repeatedly, the second and third doses produced qualitatively similar effects to the first, characterized by reductions in SysBP and DiasBP (Figs. S3, S8 and S9).

**Fig. 6:**
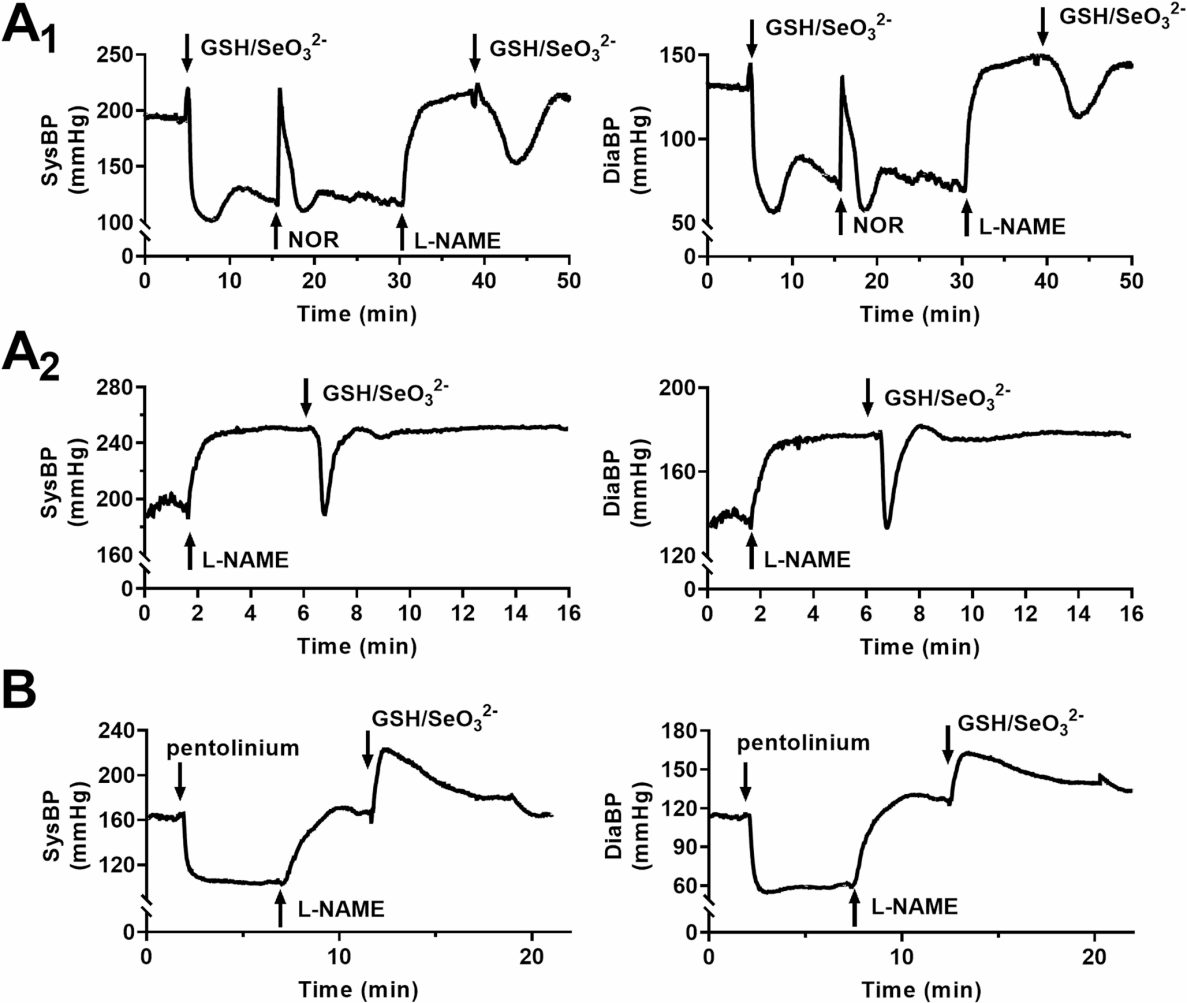
Effect of L-NAME and pentolinium on GSH/SeO_3_^2−^-induced BP changes. After the administration of GSH/SeO_3_^2−^ (75/12.5 µmol/kg), which caused BP decrease, the effects of NOR (0.5 µg/kg) and L-NAME (25 mg/kg) are shown **(A**_**1**_**)**. A repeated dose of GSH/SeO_3_^2−^ transiently attenuated the pressor effect of L-NAME. For comparison, the effect of GSH/SeO_3_^2−^ in SHR pretreated with L-NAME is shown (**A**_**2**_). The administration of pentolinium (5 mg/kg) in the sequence with L-NAME effectively diminished hypotensive effect of GSH/SeO_3_^2−^(**B**). All recordings illustrating the effects of L-NAME and pentolinium on the GSH/SeO_3_^2−^-induced modulation of BP are presented in Fig. S10

The GSH/SeO_3_^2−^ mixture failed to induce a full-strength hypotensive response when NOS was inhibited by L-NAME in SHRs (Fig. 6A), whereas NOR-mediated adrenergic signaling remained preserved. However, when pentolinium was administered in the sequence with L-NAME to exclude the contribution of sympathetic ganglionic transmission, the hypotensive response was diminished and replaced by a transient increase in blood pressure (Fig. 6B), indicating a significant role of the SNS in the hypotensive effect of the mixture.

### The Effect of GSH/SeO_3_^2−^ on APW-Ps in CAP- and LOS-Pretreated SHRs

**Fig. 7:**
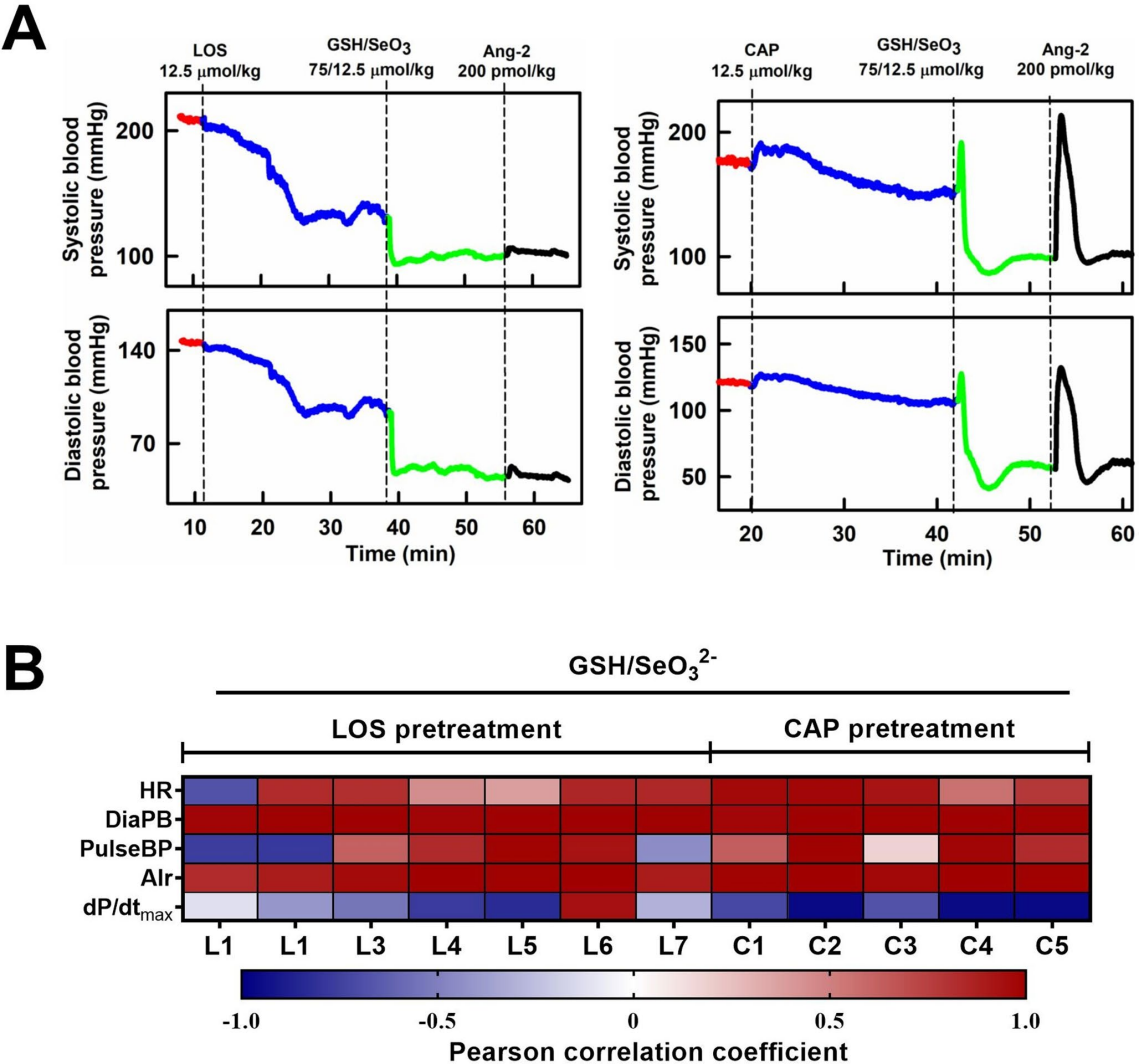
The effect of GSH/SeO_3_^2−^ on APW-Ps in SHRs pretreated with LOS or CAP. **(A)** Individual time-resolved SysBP and DiaBP records were obtained in anesthetized SHRs before (red) and after (blue) i.v. administration of LOS or CAP (12.5 µmol/kg), and following administration of the GSH/SeO_3_^2−^ (75/12.5 µmol/kg; green) and Ang-2 (200 pmol/kg; black). Vertical black dashed lines indicate the start of each administration with a duration of 15 s. **(B)** Heat map of Pearson correlation coefficients between the descending segment of SysBP and the corresponding segment of DiaBP, PulBP, AIr, HR, and d*P*/d*t*_max_ following administration of GSH/SeO_3_^2−^ in LOS (7 experiments L1-L7) and CAP pretreated (5 experiments C1-C5) SHRs. The degree of correlation is indicated by blue (negative) and red (positive), as depicted in the color scale. All correlations were statistically significant (*P* < 0.0001)

Since LOS and CAP exert their antihypertensive effects by regulating the RAAS, it was of interest to evaluate the impact of the GSH/SeO_3_^2−^ (75/12.5 µmol/kg) mixture on APW-Ps under conditions, where the RAAS was modulated by LOS or CAP. Representative time-resolved SysBP and DiaBP records demonstrated that the mixture further reduced BP after cca. 20-min long LOS (12.5 µmol/kg) or CAP (12.5 µmol/kg) pretreatment (Fig. 7A). Moreover, the correlation between SysBP and other parameters showed a similar pattern to that observed without LOS or CAP pretreatment (Fig. 7B vs. 4B). The inhibition of ATRs by LOS was verified by the use of AngII agonist. These findings indicate that the GSH/SeO_3_^2−^ mixture can further enhance the antihypertensive effects in CAP- and LOS-pretreated SHRs.

Compared with the pharmacokinetics of GSH/SeO_3_^2−^, both LOS (*n* = 7, Fig. S11) and CAP (*n* = 5, Fig. S12) required a longer time to exert their effects, as evidenced by the more pronounced negative components of the GSH/SeO_3_^2−^-induced APW-P changes during the first 10 min (Fig. S13A). As time progressed, the antihypertensive effects of CAP and LOS became fully established, whereas those of GSH/SeO_3_^2−^ gradually declined and eventually stabilized at a steady-state level. Accordingly, at the 20th min, when APW-Ps had reached steady-state levels, no significant differences were observed between treatments (Fig. S13B).

### The Vasoactive Effect of GSH+SeO_3_^2−^ Reaction Products on Isolated Arteries

**Fig. 8:**
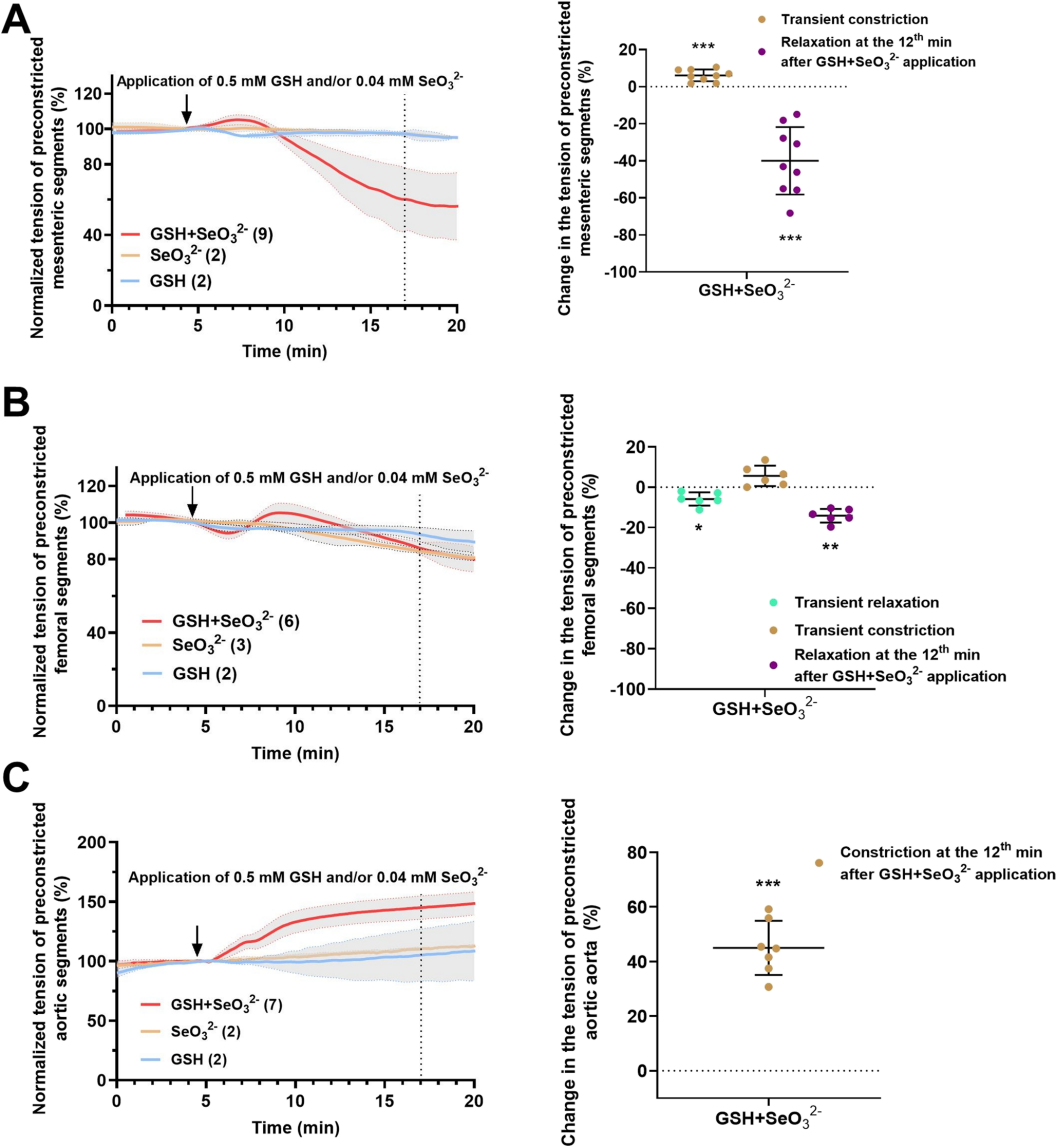
The vasoactive effect of GSH+SeO_3_^2−^ on 3 types of preconstricted SHR arteries. Arterial rings of isolated (**A**) mesenteric artery, (**B**) femoral artery and (**C**) thoracic aorta were preconstricted with NOR (1 µM), Ser (1 µM) and NOR (10 µM), respectively. Once a steady-state level was reached, GSH (0.5 mM) and subsequently SeO_3_^2−^ (0.04 mM) were applied within an interval of ~ 5–10 s. The red, blue and yellow traces in the graphs on the left represent the mean ± SD normalized change in vascular tension of the preconstricted arterial rings after application of GSH+SeO_3_^2−^, GSH and SeO_3_^2−^, respectively. The baseline represents approximately 100% and serves as the control. The graphs on the right quantify the effect of GSH+SeO_3_^2−^ on vascular tension calculated as the change in tension (% of baseline) of the preconstricted artery at indicative points (∆*T*/*T*_0_ × 100%, where ∆*T* is the difference between baseline tension *T*_0_ and actual tension *T* obtained at the indicative point). The indicative points include inflection points and endpoints at the 12th min. The data are presented as individual values with means ± SD, with the number of experiments indicated in parentheses. Statistics: a one-way ANOVA with repeated measures followed by Dunnett’s multiple comparisons test (**A**) and (**B**), or a Student´s t-test (**C**) was used: *P < 0.05 vs. control, **P < 0.01 vs. control, ***P < 0.001 vs. control (statistical results are presented in Tab. S2)

We evaluated vascular reactivity of three types of arteries: thoracic aorta, femoral and mesenteric arteries isolated from SHRs (Fig. 8). Interestingly, the reactivity profiles of all three artery types were similar to those observed in normotensive rats when using the same molar ratio of GSH+SeO_3_^2−^ (0.5/0.04 mM, 12.5:1) [18]. The notation GSH+SeO_3_^2−^ is used to indicate the sequential application of GSH and SeO_3_^2−^. Mesenteric arteries preconstricted with NOR exerted a biphasic response to GSH+SeO_3_^2−^. An initial short phase of increased vascular tension was followed by a relaxation phase with vascular tension stabilization at the end. On average, a 40% relaxation was observed by the 12th min after GSH+SeO_3_^2−^ application. Neither GSH nor SeO_3_^2−^ applied individually significantly affected mesenteric artery tension. The femoral arteries showed a more complex response to GSH+SeO_3_^2−^. An initial significant decrease in tension was followed by a transient constriction, which gradually progressed into a long-lasting relaxation. However, the slow relaxation phase was also observed in the femoral artery with SeO_3_^2−^ and partially with GSH. In contrast, the thoracic aorta exhibited a continuously increasing vascular tension in response to GSH+SeO_3_^2−^. Partial constriction was observed with the individual applications of GSH and SeO_3_^2−^, but neither reached the level of GSH+SeO_3_^2−^-induced constriction. These results suggest that the vasoactivity of GSH+SeO_3_^2−^ strongly depends on arterial size, with vasorelaxation observed in the smallest arteries.

**Fig. 9:**
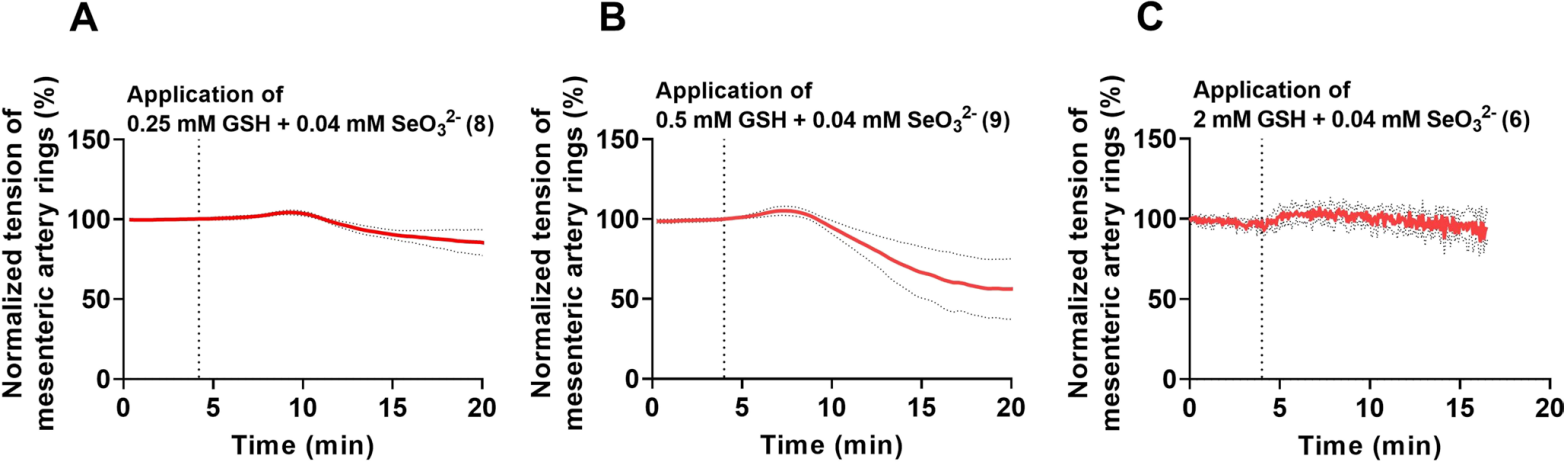
Vascular reactivity of preconstricted mesenteric arteries to varying GSH:SeO_3_^2−^ molar ratios. The vasorelaxation effect of GSH/SeO_3_^2−^ on mesenteric arteries isolated from SHRs was evaluated at a fixed SeO_3_^2−^ concentration of 0.04 mM with increasing concentrations of GSH: 0.25 mM (**A**); 0.5 mM (**B**); and 2mM (**C**) GSH. Traces represent the mean ± SD changes in vascular tension, with the number of experiments indicated in parentheses. The baseline tension of preconstricted mesenteric artery (100%) was used as the control reference

The outcome of the reaction between GSH and SeO_3_^2−^ is determined by the molar ratio of reactants [26]. To address this, we tested mesenteric artery reactivity to GSH/SeO_3_^2−^ at two additional concentrations of 2/0.04 mM and 0.25/0.04 mM, corresponding to molar ratios of 50:1 and 6.25:1, respectively (Fig. 9). When the concentration of GSH was either higher or lower than 0.5 mM, the vasorelaxant effect was reduced. Moreover, the onset of the vasomotor response depended on GSH concentration.

### Factors Underlying the Vasoactive Effects of GSH+SeO_3_^2−^

**Fig. 10:**
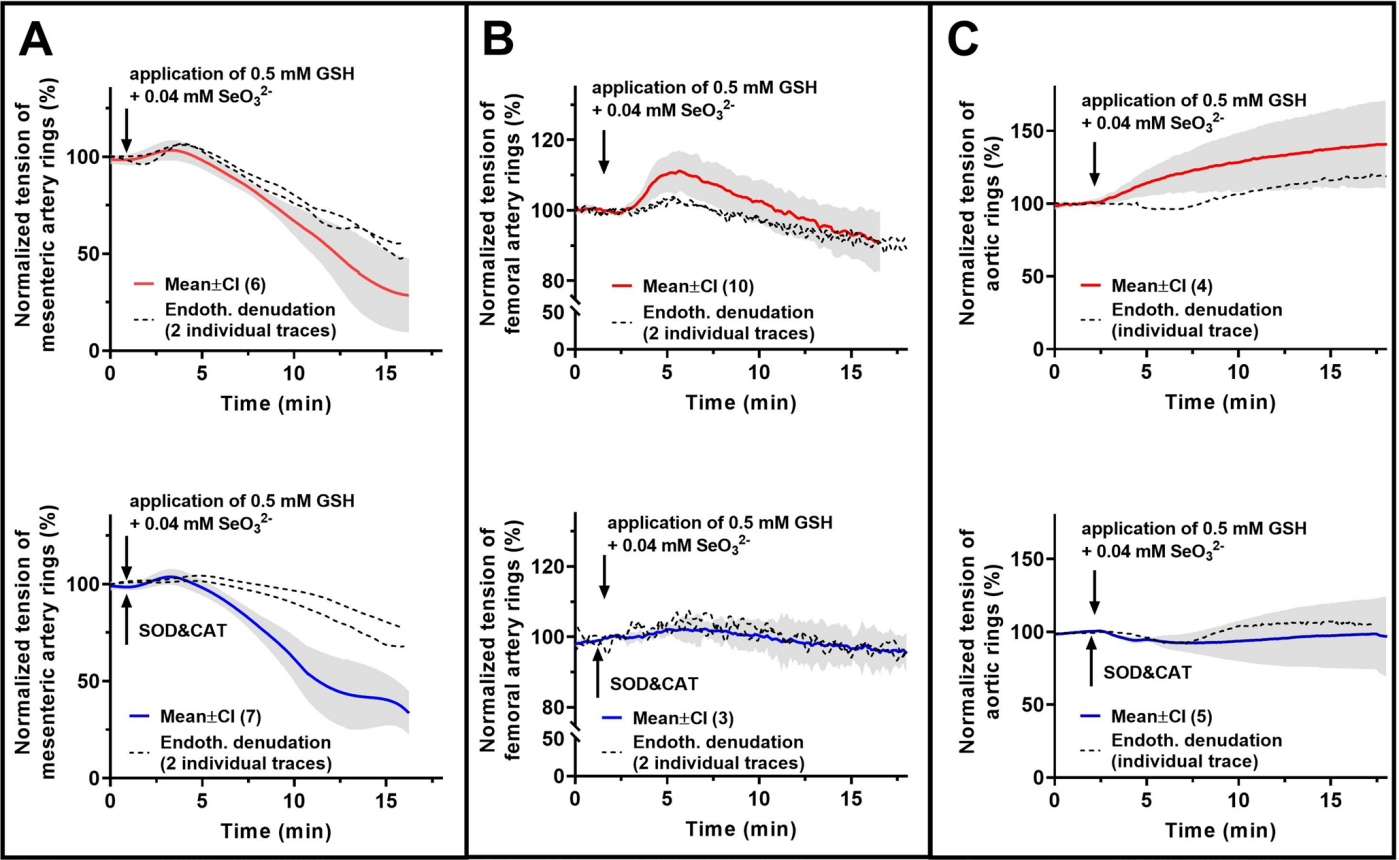
Effect of SOD and CAT on arterial responses to GSH+SeO_3_^2−^. Isolated rings of mesenteric artery (**A**), femoral artery (**B**) and thoracic aorta (**C**) were preconstricted with NOR (1 µM), Ser (1 µM) and NOR (10 µM), respectively. Once a stable plateau was reached (defined as 100% tone), GSH (0.5 mM) and subsequently SeO_3_^2−^ (0.04 mM) were applied to the organ bath, either in the presence of SOD and CAT (both with activity > 110 U/mL; lower panels, blue traces) or in their absence (upper panels, red traces). Traces represent the mean changes in vascular tension, with the grey-shaded are indicating the 95% confidence interval (CI).The number of experiments is indicated in parentheses. Black dotted lines indicate individual responses to GSH+SeO_3_^2−^ in endothelial-denuded arterial rings. All recordings are provided in Fig. S15

H_2_O_2_ functions as an important endothelium-derived hyperpolarising factor (EDHF) that mediates the vasorelaxation in mesenteric arteries [27], therefore its generation during GSH and SeO_3_^2−^ reaction may be relevant in the regulation of vascular tone. We confirmed that exogenous H_2_O_2_ induced relaxation in mesenteric arteries of SHRs, and this effect was abolished by CAT (Fig. S14). Therefore, we used a combination of SOD and CAT to exclude the role of ^●^O_2_^−^/H_2_O_2_ in the vasoactive response to GSH+SeO_3_^2−^. Interestingly, SOD and CAT either prevented or markedly reduced constriction in aortic and femoral artery rings, while they had no effect on vasomotor response in mesenteric arteries (Fig. 10). This observation is consistent with finding in non-preconstricted aortic rings, where similar reduction in vascular constriction was detected in the presence of SOD and CAT (Fig. S15D). To further substantiate our findings in SHRs, we used Wistar rats as a normotensive model to investigate the vasomotor effects of GSH+SeO_3_^2−^ and to assess the contribution of ^●^O_2_^−^ to this response. Compared with SHRs, GSH+SeO_3_^2−^ induced a more pronounced relaxation of mesenteric arteries in Wistar rats (Fig. S16). Notably, this relaxation was diminished upon co-application with SOD, indicating a greater modulatory role of ^●^O_2_^−^ in the normotensive model than in SHRs.

Next, we investigated the role of the endothelium in the observed vasoactive effects. Endothelial denudation significantly attenuated, but did not abolish, GSH+SeO_3_^2−^-induced relaxation in mesenteric arteries from SHRs (Fig. 10), indicating the involvement of both endothelium-dependent mechanisms and direct effects on vascular smooth muscle cells (VSMCs). The GSH+SeO_3_^2−^-induced relaxation following endothelial denudation was likewise preserved in mesenteric arteries from Wistar rat (Fig. S16). Consistent findings were obtained in the femoral artery and aorta, where the vasomotor responses to GSH+SeO_3_^2−^ persisted after endothelial denudation, with attenuation of the constrictive phase observed in the absence of SOD and CAT.

For pharmacological characterization using L-NAME, glibenclamide, and TEA, only the mesenteric artery and aorta were selected (Fig S17). Due to the limited number of experiments, the robustness of physiological interpretation is constrained. In all cases, mesenteric arteries exhibited relaxation in response to GSH+SeO_3_^2−^, although L-NAME produced a modest attenuation observed at 12 min. In the thoracic aorta, L-NAME reduced the constrictive phase in the absence of SOD and CAT. Interestingly, glibenclamide appeared to markedly decrease vascular tone in aortic rings in the presence of SOD and CAT. Overall, none of the pharmacological modulators eliminated vascular reactivity to GSH+SeO_3_^2−^. However, L-NAME partially recapitulated the effects of endothelial denudation, supporting the contribution of endothelium-dependent factors in mediating these vasoactive responses.

### Spectrophotometric and Reducing Properties of GSH/SeO_3_^2−^

**Fig. 11:**
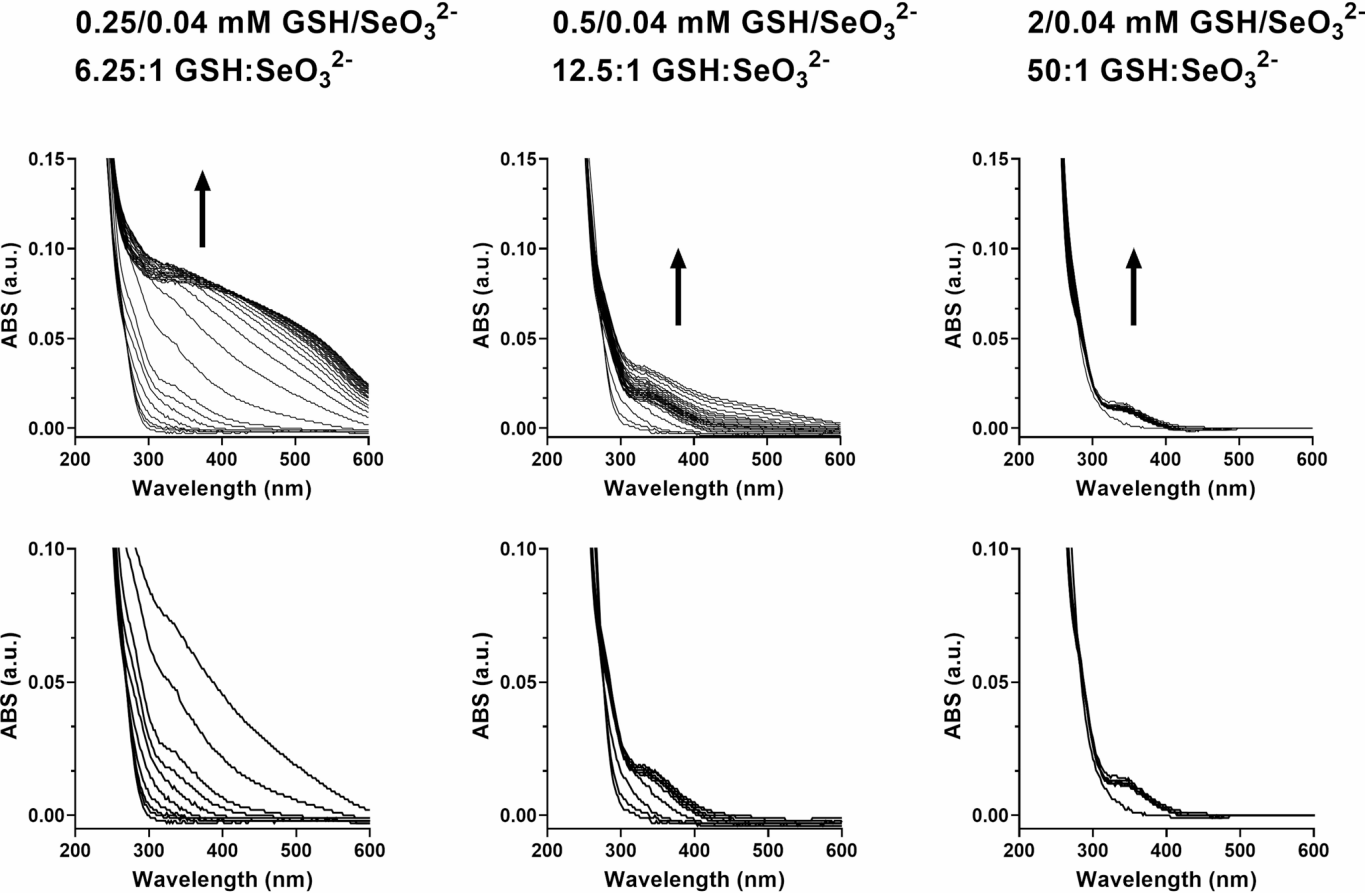
Spectrophotometric properties of GSH/SeO_3_^2−^ at 3 different molar ratios. Time-resolved UV-VIS spectra of GSH/SeO_3_^2−^ at concentrations of 0.25/0.04 mM, 0.5/0.04 mM, and 2/0.04 mM were recorded in Krebs buffer at 37 ± 1 °C every 20 s for 10 min. A 35 µL aliquot of GSH (stock solutions of 5, 10, 40 mM prepared in ddH_2_O) were added to 665µL of Krebs buffer containing 0.04 mM SeO_3_^2−^ (corresponding to 20 µL of a 1.4 mM stock solution prepared in ddH_2_O). The upper panel presents spectra collected over the entire 10-min period, whereas the lower panel shows only the first 10 spectra to facilitate comparison of ABS progression at the onset of the reaction. An arrow indicates the direction of spectral acquisition

The different GSH: SeO_3_^2−^ molar ratios of 6.25:1, 12.5:1 and 50:1 elicited distinct vasoactive responses in the mesenteric artery (Fig. 9). Vasoconstriction was observed at all three ratios, with a decrease in GSH delaying its onset, whereas relaxation was occurred at the 6.25:1 and 12.5:1 ratio. Correlation between the vasoactive responses and UV-VIS spectra of the GSH/SeO_3_^2−^ reaction at various molar ratios showed an important role of Se^0^ formation. The lowest GSH concentration was associated with a fast increase in the ABS signal across a broad wavelength range, indicating massive Se^0^ formation followed by organization into colloidal particles responsible for the observed turbidity (Fig. 11). An ABS peak around 350 nm (ABS_350_) was observed at all ratios and persisted for a varying duration. At the 50:1 ratio, ABS_350_ increased rapidly and remained stable over the 10-min experiment, whereas at the 12.5:1 and particularly 6.25:1 ratio, ABS_350_ disappeared in the broader spectrum, indicating increased turbidity. When SOD and/or CAT were co-applied with GSH/SeO_3_^2−^ (0.5/0.04 mM), the intensity in a broad wavelength range significantly decreased, suggesting reduced colloidal Se^0^ formation (Fig. S18). The contribution of ROS to the formation of colloidal Se^0^ was also visually evident (Fig S19).

UV-VIS spectra of the interaction of GSH (150 mM) with SeO_3_^2−^ (25 mM) in vivo were also detected (Fig. S20A). A spectrum of GSH or SeO_3_^2−^ alone showed no ABS above 300 nm and remained stable for up to 3 min. However, the GSH/SeO_3_^2−^ mixture produced a significant ABS signal between 300 and 400 nm within 20–60 s after preparation. Subsequently, the ABS extended across the entire monitored range (230–900 nm), indicating the formation of turbidity-producing species, whose concentration continued to increase. These spectrophotometric measurements were consistent with the visual color change of the GSH/SeO_3_^2−^ mixture during the 20-min incubation period (Fig. S1). To evaluate the reducing properties of the mixture as they developed over time, the GSH/SeO_3_^2−^ (150/25 mM) mixture was incubated up to 30 min at 24 ± 1 °C (Fig. S20 B and C). After a 1,000-fold dilution to 150/25 µM, the mixture effectively reduced the stable ^●^cPTIO radical (100 µM), confirming the generation of reducing species. However, its reducing capacity declined after incubation periods longer than 5 min.

### pDNA Cleavage Induced by GSH/SeO_3_^2−^

**Fig. 12:**
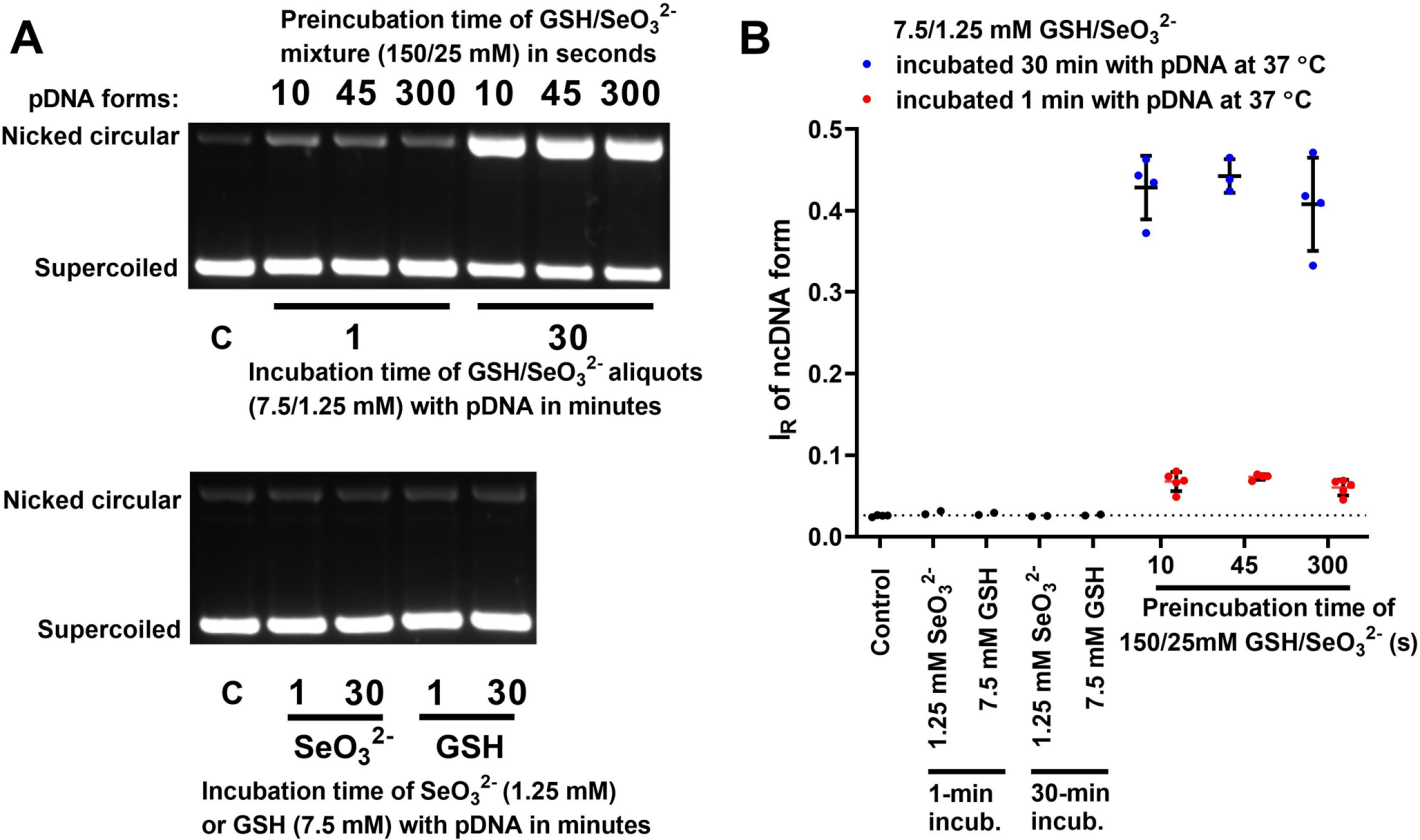
The effect of incubation time on pDNA cleavage induced by GSH/SeO_3_^2−^. The impact on pDNA cleavage was analyzed considering two factors: the preincubation period of the GSH/SeO_3_^2−^ mixture itself (10, 45 and 300 s) and the incubation period of GSH/SeO_3_^2−^ with pDNA (1 and 30 min). (**A**) A representative gel illustrates the cleavage of pDNA induced by GSH (7.5 mM), SeO_3_^2−^ (1.25 mM) and their mixture after preincubation for 10, 45 or300 s. The reaction mixture was prepared as described for in vivo experiments. Stock solutions of 200 mM GSH and 100 mM SeO_3_^2−^ were mixed at a 3:1 ratio to yield final concentrations of 150 and 25 mM, respectively. Then, 1 µL aliquot was added to 19 µL of pDNA solution (containing 0.2 µg of pDNA in 25 mM sodium phosphate buffer and 50 µM DTPA) and incubated for either 60 s or30 min at 37 ± 1 °C. In the gel, the bands at the bottom and top correspond to the circular supercoiled and nicked circular (ncDNA) forms of pDNA, respectively. (**B**) Quantitative analysis of pDNA cleavage was performed to estimate the pDNA cleavage potency of the mixture. The control sample received no treatment. The relative intensity (I_R_) of the ncDNA form are presented as individual values with means ± SD

Our previous work demonstrated that the GSH/SeO_3_^2−^ mixture effectively cleaves pDNA, exhibiting a bell-shaped dependence on the molar ratio [18]. Based on aforementioned data, we assumed that cleavage would occur at a 6:1 (GSH: SeO_3_^2−^) molar ratio after a 30-min incubation period with pDNA. However, the impact of significantly shorter incubation times on pDNA integrity remained unclear. To address this, a reaction mixture containing 150 mM GSH and 25 mM SeO_3_^2−^ was preincubated for 10, 45 and 300 s at 24 ± 1 °C before being added to the pDNA solution. After addition, a final concentration of 7.5/1.25 mM (GSH/SeO_3_^2−^) was achieved in the pDNA solution, which was further incubated for either 60 s or30 min at 37 ± 1 °C (Fig. 12). Consistent with previous findings [18], significant pDNA damage was observed after the 30-min incubation period. In contrast, the shorter 60-s incubation period with pDNA led to a significantly less efficient cleavage, even when the GSH/SeO_3_^2−^ reaction was preincubated for 300 s. Notably, the extent of pDNA cleavage appeared to remain unaffected by preincubation periods ranging from 10 to 300 s.

## Discussion

In this study, we demonstrate that the products of the GSH/SeO_3_^2−^ mixture reduce BP in SHRs to a greater extent than previously observed in normotensive rats [18]. Furthermore, the reaction generates vasoactive species exerting effects that are at least partially endothelium-independent. These findings indicate that the products generated during SeO_3_^2−^ reduction, either through its reaction with GSH in the mixture or with reducing agents present in the bloodstream, may serve as effective antihypertensive agents, even under conditions of endothelial dysfunction.

The interaction of SeO_3_^2−^ with GSH is complex and involves multiple reaction steps, generating numerous (intermediate) products, such as GSSeSG, RSSe^−^, Se^0^, and Se^2−^ as well as non-selenium reactive species, such as ^●^O_2_^−^. The following reactions have been proposed based on earlier studies [26, 28]:

1 $$\\\begin{array}{c}SeO_{3}^{2-}\;+\;4GSH\;+\;2H^{+\;}\rightarrow\;GSSeSG\;+\;GSSG\;+\;3H_2O\end{array}$$

2 *GSSeSG+GSH→ GSSeH+GSSG*

3 $$GSSeH\rightarrow Se^0+GSH$$

4 $$GSSeH+GSH\rightarrow H_2Se+GSSG$$

5 $$H_2Se+2O_2\rightarrow Se^0+2^{\bullet} O_2^-+2H^+$$

6 $$\\\begin{array}{c}H_2Se+1/2O_2+2GSH\rightarrow H_2Se+GSSG+H_2O\end{array}$$

The reactions outlined above demonstrate that Se(IV) in SeO_3_^2−^ undergoes stepwise reduction, producing a spectrum of Se-containing products. This gradual reduction explains the delayed effect of SeO_3_^2−^ on APW-Ps after i.v. administration compared to GSH/SeO_3_^2−^, highlighting the necessity for SeO_3_^2−^ to be “activated” through reduction in the bloodstream. When administered intravenously to rats, SeO_3_^2−^ is rapidly and selectively taken up by RBCs, where it is reduced by GSH to form Se^2−^, which is released into the plasma and subsequently binds to albumin [29]. While plasma GSH concentrations (2 − 20 µM) are relatively low compared to intracellular levels in RBCs (1.2 − 1.5 mM) [30], other members of plasma redox pool, such as cysteine, can potentially reduce SeO_3_^2−^ to biologically active Se forms. Our previous work has shown that the in vitro interaction between SeO_3_^2−^ and cysteine generates reactive species with properties qualitatively similar to those generated through the SeO_3_^2−^/GSH interaction [18]. It should also be emphasized that the reduction process is governed by the availability of GSH and the GSH:SeO_3_^2−^ ratio plays a critical role in determining the resulting products and, consequently, their biological effects. In addition, the GSH: SeO_3_^2−^ interaction ratio also determines the bell-shaped production of ^●^O_2_^−^ [31]. Considering these associations, the reducing agent: SeO_3_^2−^ ratio may explain the dose-dependent effects of SeO_3_^2−^ on BP, as previously reported − lower doses exhibit hypertensive responses, whereas higher doses produce hypotensive effects [32, 33]. We previously reported that pDNA cleavage exhibits a bell-shaped dependence on the thiol:SeO_3_^2−^ ratio after a 30-min incubation with pDNA [18]. In the present study, the products generated in the SeO_3_^2−^/GSH mixture within the first 300 s had a comparable pro-cleavage activity, and even a 1-min incubation with pDNA was sufficient to produce detectable pDNA damage. Accordingly, the mixture incubated for 45 s and subsequently administered into SHRs contained active species capable of exerting detrimental effects on pDNA. We further demonstrate that the GSH: SeO_3_^2−^ molar ratio determines the vasomotoric response in mesenteric arteries, as discussed below.

Interestingly, the mixture, but not GSH or SeO_3_^2−^ alone, induced relaxation in mesenteric artery isolated from SHRs. This finding further supports that SeO_3_^2−^ requires a reducing agent, such as GSH, to generate bioactive species capable of altering vascular tone. The kinetics of the reaction between GSH and SeO_3_^2−^ was reflected in the physiological response time. In the in vivo experiments, the GSH/SeO₃²⁻ mixture was pre-incubated for 45–50 s before administration, allowing formation of reactive intermediates prior to entering the circulation. In contrast, in the ex vivo setting, GSH and SeO₃²⁻ were applied sequentially to isolated vessels, requiring additional time for in situ generation of biologically active species. Accordingly, the vasomotor responses developed more slowly in isolated vessels. A similar delay was evident following i.v. administration of SeO₃²⁻ alone compared with the preformed GSH/SeO₃²⁻ mixture, further supporting the importance of a reducing biological environment and the role of reaction kinetics in determining the onset of the physiological response.

The mesenteric artery relaxation depended on the GSH: SeO_3_^2−^ molar ratio. Relaxation was diminished when the ratio was shifted from 12.5:1 to either 6.25:1 or 50:1. At the higher GSH concentration, the relaxation was almost completely abolished, whereas at the lower ratio relaxation was still retained to some extent. By comparing the observed vasoactive effects with the UV-VIS spectra recorded at different GSH: SeO_3_^2−^ molar ratios, we propose that Se^0^ formation is associated with the mesenteric artery relaxation. Under conditions of GSH excess, GSSeH can be further reduced to H_2_Se, which remains protected from oxidation by O_2_ (Eqs. 4 and 6). When GSH availability is limited, GSSeH undergoes dismutation to Se^0^ (Eq. 3), which can subsequently be organized into Se^0^ nanoparticles in a concentration-dependent manner. Se^0^-containing nanoparticles without stabilization can readily undergo aggregation [34]. The change in Se^0^ nanoparticles size determines their biological properties, as Se^0^ nanoparticles have been reported to exhibit size-dependent free radical scavenging and redox activity [35, 36].

Based on UV-VIS spectrophotometric data, the highest level of turbidity was observed at the molar ratio of 6.25:1 (GSH: SeO_3_^2−^). This indicates that an increased amount of SeO_3_^2−^ promoted enhanced colloidal Se^0^ formation, followed by its aggregation and subsequent sedimentation into inactive forms. These processes explain the attenuated relaxation observed in the mesenteric artery compared with the 12.5:1 GSH: SeO_3_^2−^ ratio. Notably, the presence of SOD and/or CAT decreased the formation of colloidal Se^0^ during the interaction between GSH and SeO_3_^2−^ (0.5/0.04 mM), as evidenced by reduced turbidity in the UV-VIS absorption spectra. This finding underlies the role of ^●^O_2_^−^/H_2_O_2_ in modulating the reaction outcome. Consistent with this, we also observed that SOD attenuated mesenteric artery relaxation in Wistar rats. However, SOD + CAT had no effect on the relaxation response in SHRs. This divergence between SHRs and Wistar rats is likely attributable to the combination of two major factors: (i) distinct (patho)physiological characteristics of the animal models used, and (ii) the above-mentioned influence of ^●^O_2_^−^/H_2_O_2_ on the formation of Se^0^ and/or colloidal Se^0^-containing species. The involvement of the first factor is supported by our finding that GSH+SeO_3_^2−^-induced relaxation in NOR-preconstricted mesenteric arteries after 10 min reached 64.0% (± 10.6, *n* = 6) in Wistar rats, but only 33.2% (± 7.4, *n* = 6) in SHRs. In comparison, WKY rats exerted an 81.5% (± 8.1, *n* = 6) decrease in mesenteric artery tension, as we previously reported [18]. The substantially attenuated response to GSH+SeO_3_^2−^ observed in SHRs, relative to normotensive strains, likely reflects hypertension-associated vascular alterations. Reduced sensitivity to vasorelaxants in SHRs compared to WKY rats has been reported elsewhere, involving both endothelium-dependent and endothelium-independent characteristics [37, 38]. The second factor considers that the formation of Se^0^ played a significant role in the vasorelaxation of the mesenteric artery. The SOD-mediated inhibition of colloidal Se^0^ formation likely decreased the vasorelaxation response in mesenteric arteries isolated from Wistar rats. However, in SHRs, the reduced vasomotor sensitivity to GSH+SeO_3_^2−^ probably precluded detection of any attenuation of vasorelaxation resulting from SOD-mediated suppression of Se^0^ formation.

In summary, vasorelaxation induced by GSH+SeO_3_^2−^ in the mesenteric arteries of SHRs depended on the GSH: SeO_3_^2−^ molar ratio but was independent of SOD + CAT. Notably, this relaxation was preceded by a transient constriction of small amplitude, which persisted across different GSH: SeO_3_^2−^ ratios and remained unaffected by the presence of SOD + CAT. This initial constrictive response may reflect the effects of intermediate products formed prior to Se^0^ generation. It should be noted that our conclusions rely on the correspondence between the reported reaction mechanisms of SeO_3_^2−^ reduction by GSH, the observed UV-VIS spectra, and the vasomotor responses to GSH: SeO_3_^2−^.

In contrast, GSH+SeO_3_^2−^ elicited pronounced vasoconstriction in the thoracic aorta. The vasomotor response in the femoral artery was more complex, consisting of a brief, mild relaxation followed by a moderate vasoconstriction and a subsequent return to relaxation. Interestingly, SOD + CAT abolished the responses in these large vessels, unlike in smaller vessels such as the mesenteric artery, indicating a critical involvement of ^●^O_2_^−^/H_2_O_2_. Thus, vasomotor responses to GSH+SeO_3_^2−^ varied across arterial beds, highlighting the heterogeneity of vascular tone regulation mediated by redox-active species.

These findings are consistent with our previous report on the vasoactive effects of phthalic selenoanhydride (R-Se) in arteries isolated from Wistar rats, which showed the following concentration-dependent sensitivity: mesenteric artery > renal artery > femoral artery > thoracic aorta [22]. Assuming that H_2_Se released from R-Se is readily oxidized by O_2_ to Se^0^ (Eq. 5), GSH+SeO_3_^2−^-mediated relaxation may exhibit a vascular bed-dependent pattern similar to that observed for R-Se.

As noted earlier, ROS also directly modulate vascular tone, as demonstrated for H_2_O_2_ − the major component of EDHF [39]. The contribution of EDHF to vasorelaxation varies along the arterial tree, with increasing importance in smaller-radius vessels, such as resistance arteries [40]. Although we confirmed that exogenous H_2_O_2_ induced relaxation in the mesenteric arteries from SHRs, CAT did not inhibit GSH+SeO_3_^2−^-induced relaxation, thereby excluding H_2_O_2_ as its mediator (at least at the levels produced). In contrast, the vasorelaxation in larger conduit vessels is predominantly mediated by NO signaling. However, it have been reported that increased ^●^O_2_^−^ formation from NADP(H) oxidase in aortas from SHR contributed to the vasoconstrictor responses and counteracted NO-modulated responses [41]. This interpretation explains the strong constriction observed in the thoracic aorta following GSH+SeO_3_^2−^, which was abolished by SOD + CAT. In conclusion, the vasomotoric response to GSH+SeO_3_^2−^ in larger vessels involves ROS produced during the interaction between GSH and SeO_3_^2−^.

However, direct measurements of ^●^O_2_^−^/H_2_O_2_ were not performed in the present study, and the interpretation of ROS-mediated vasomotor responses is therefore derived primarily on the scavenging effects of SOD and CAT on these ROS. Nevertheless, the involvement of ROS should be interpreted with caution, as ^●^O_2_^−^/H_2_O_2_ may also participate in the redox cycling of selenium intermediates in the presence of GSH [42]. Consequently, SOD and CAT may alter the equilibrium of reactive intermediates formed during the GSH+SeO_3_^2−^ reaction, as indicated by the UV-Vis data.

On the other hand, the acute toxicity of SeO_3_^2−^ is primarily attributed to ROS generation and the induction of oxidative stress. In addition to their role as vasomodulators, the potential contribution of ^●^O_2_^−^/H_2_O_2_ to toxic effects under ex vivo conditions was delineated using SOD and CAT. In the case of i.v. administration of the preformed GSH/SeO_3_^2−^ mixture, UV–Vis spectroscopic analysis demonstrated that reduction of SeO_3_^2−^ by GSH progressively leads to the formation of colloidal Se^0^. Recent studies indicate that Se^0^-containing species represent the least toxic selenium forms, potentially making them more favorable than SeO_3_^2−^ itself [17].

In the mesenteric artery, GSH+SeO_3_^2−^-induced relaxation was only partially reduced by endothelium removal or L-NAME preincubation, indicating involvement of both endothelium-dependent and endothelium-independent mechanisms. In the thoracic aorta, the GSH+SeO_3_^2−^-induced constriction was partially diminished by endothelial removal and by NOS inhibition with L-NAME. These findings suggest that the aortic constriction was mediated by an endothelium-derived contracting mechanism that depends on both NO and ROS. Besides decreasing NO bioavailability, ^●^O_2_^−^ reacts with NO to form peroxynitrite, which significantly impairs both endothelium-dependent and -independent aortic relaxation in SHRs and WKY rats [43].

Mesenteric arteries, as resistance vessels, are major determinants of peripheral vascular resistance; their dilation decreases peripheral resistance and consequently lowers BP. Administration of SeO_3_^2−^ and GSH/SeO_3_^2−^ decreased SysBP, DiaBP, PulBP, Air, and HR, while increasing d*P*/d*t*_max_. However, the effects observed in organ bath experiments reflect more direct consequences of the GSH+SeO_3_^2−^ reaction, whereas i.v. administration of the GSH/SeO_3_^2−^ mixture introduces additional layers of complexity. These include not only blood-associated factors, such as redox buffering and antioxidant capacity, which influence Se disproportionation and ROS scavenging, but also systemic mechanisms of blood pressure regulation as well as. This aspect was demonstrated by the significant contribution of the SNS to the hypotensive effects of the GSH/SeO_3_^2−^ mixture.

Recent evidence suggests that wave reflection indices like augmentation index (AI) are influenced not only by conduit-artery stiffness but also by small artery tone and microvascular resistance [44]. Furthermore, the velocity of left ventricle contraction can affect AI as much as increased arterial stiffnes [45] and strong negative correlations between HR and AI have been reported [46]. Herein, AI in SHRs was significantly higher than that obtained in normotensive rats [18] and decreased along with HR after the administration of GSH/SeO_3_^2−^. In contrast to other hemodynamic parameters, arterial d*P*/d*t*_max_ was elevated and remained also high after BP stabilized at the steady-state levels. Arterial d*P*/d*t*_max_ reflects both central and peripheral factors, and a significant relationship between arterial d*P*/d*t*_max_ and LV contractility has been reported [47]. Consequently, arterial d*P*/d*t*_max_ is considered an indicator of LV contractility under varying loading and inotropic conditions [48]. Thus, the observed increase in d*P*/d*t*ₘₐₓ may result either from a direct effect of the GSH/SeO₃²⁻ mixture on myocardial contractility or from a reflexive enhancement of sympathetic activity triggered by BP lowering.

Moreover, GSH/SeO_3_^2−^ effectively reduced BP even in SHRs pretreated with two commonly used RAAS-trageting antihypertensive drugs, LOS and CAP. However, it should be noted that ISO anesthesia in SHRs influences the systems involved in the BP regulation, as ISO increases participation of RAAS, while suppresses the contribution of sympathetic nervous system to BP maintenance [49]. Despite this effect, our findings appear particularly relevant because current antihypertensive therapies targeting the RAAS or sympathetic nervous system are ineffective in approximately 40% of patients [50]. Therefore, further pharmacological studies can clarify the impact of GSH/SeO_3_^2−^ on cardiovascular system and its potential in antihypertensive therapy.

## Conclusion

The reduction of SeO_3_^2−^ by GSH generates physiologically active species that lower blood pressure, heart rate, and augmentation index in SHRs. We propose that observed hypotensive effects result from inhibition of sympathetic nervous system activity and a reduction in peripheral vascular resistance. The formation of Se^0^ and/or colloidal Se^0^ likely mediates vasorelaxation in small arteries through mechanisms that are at least partially endothelium-independent. However, additional intermediates formed during the GSH/SeO_3_^2−^ reaction may also contribute as vasoactive mediators. Furthermore, the additive antihypertensive effects observed in SHRs pretreated with LOS and CAP suggest that GSH/SeO_3_^2−^-genereted species may provide an additional layer of therapeutic efficacy.

## Supplementary Information

Below is the link to the electronic supplementary material.

Supplementary Material 1 (PDF 468 KB)

Supplementary Material 2 (DOCX 6.51 MB)

## Data Availability

All findings and conclusions are based on the presented figures in the main text or in the Supplementary Materials. The datasets used and/or analyzed are available from the corresponding author on reasonable request.
