# Supplementary material for: Glutathione/Selenite Interaction Products as Potent Vascular Smooth Muscle Tone Modulators with Antihypertensive Effects in Spontaneously Hypertensive Rats
Source: Biol Trace Elem Res. 2026 Jun 24;204(9):7301–19. doi: 10.1007/s12011-026-05188-1 (PMC13437607; doi:10.1007/s12011-026-05188-1)
Supplement: Supplementary file 1 [file 12011_2026_5188_MOESM1_ESM.pdf]

Biological Trace Element Research

A list of original pDNA gels analyzed in the manuscript entitled

Glutathione/selenite interaction products as potent vascular smooth muscle tone modulators with antihypertensive effects in spontaneously hypertensive rats

Lenka Tomasova [1], Peter Balis [2], Marian Grman [1], Anna Zemancikova [2], Sona Cacanyiova [2], Miroslav Chovanec [3], Karol Ondrias [1], Anton Misak [1,*]

* Correspondence: anton.misak@savba.sk

The gel images presented here were used to quantify pDNA cleavage, as shown in Figure 13.

The pDNA gels show the supercoiled (sc) and nick circular (nc) forms of pDNA following exposure to the GSH/selenite mixture. Initially, 150 mM GSH and 25 mM SeO_3^{2-} was mixed at a 3:1 ratio and preincubated for 10, 45, or 300 s at 24±1 °C. Subsequently, an aliquot of this mixture was added to the pDNA solution to attain final concentrations of 7.5 mM GSH and 1.25 mM SeO_3^{2-}, followed by further incubation for 1 or 30 min at 37°C.

Apteryx symbols indicate different incubation periods of the mixture with pDNA.
* marks a 1-min incubation
** marks a 30-min incubation

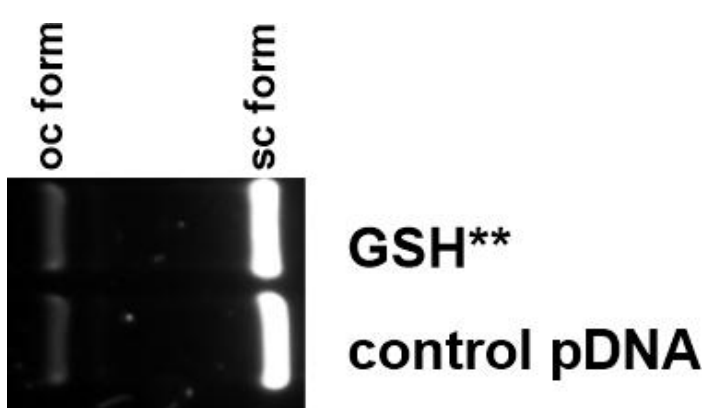

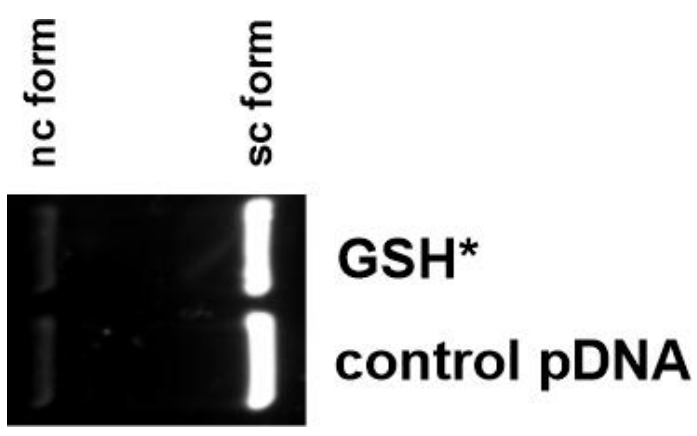

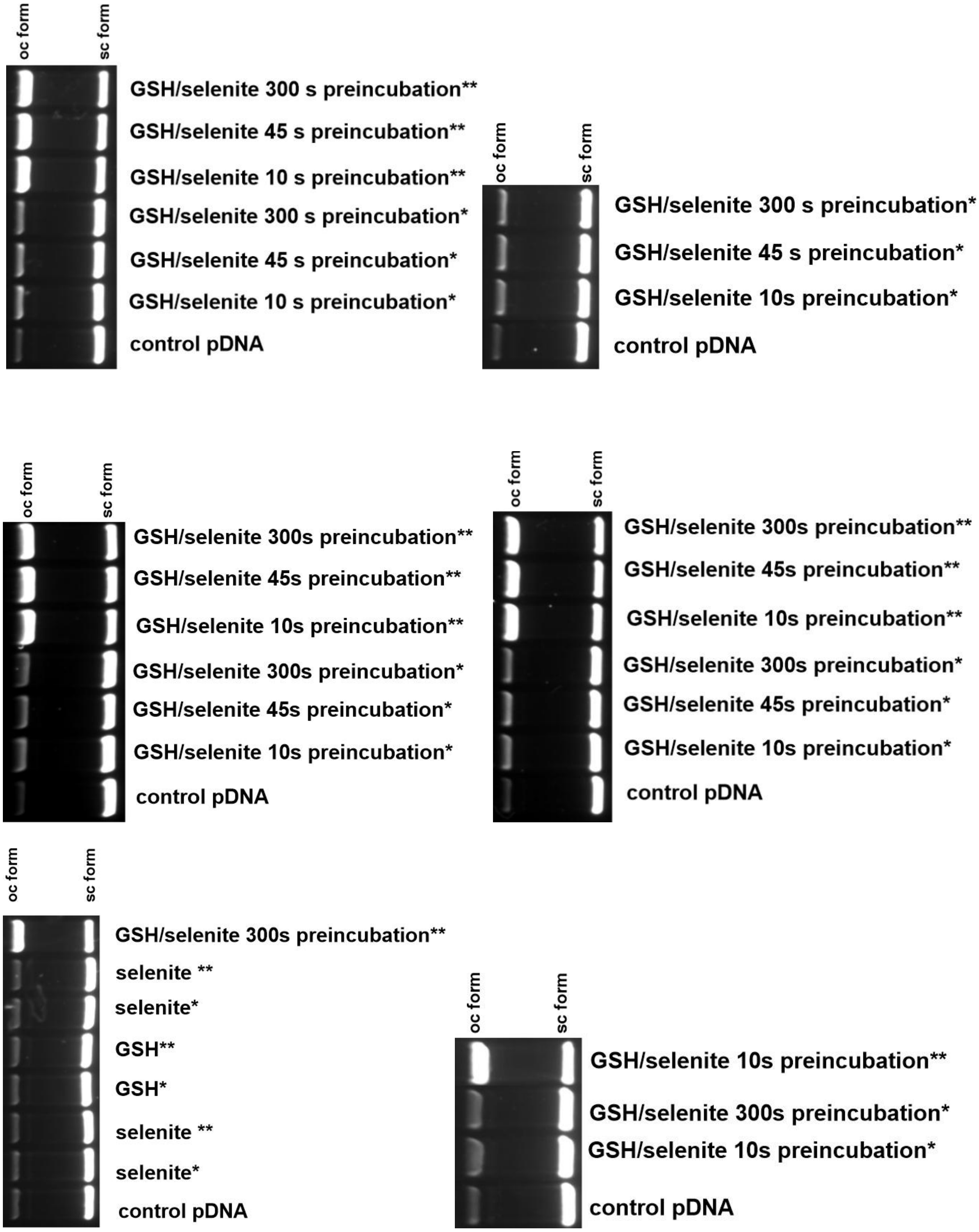
oc form
sc form
GSH/selenite 300 s preincubation**
GSH/selenite 45 s preincubation**
GSH/selenite 10 s preincubation**
GSH/selenite 300 s preincubation*
GSH/selenite 45 s preincubation*
GSH/selenite 10 s preincubation*
control pDNA
oc form
sc form
GSH/selenite 300 s preincubation*
GSH/selenite 45 s preincubation*
GSH/selenite 10s preincubation*
control pDNA
oc form
sc form
GSH/selenite 300s preincubation**
GSH/selenite 45s preincubation**
GSH/selenite 10s preincubation**
GSH/selenite 300s preincubation*
GSH/selenite 45s preincubation*
GSH/selenite 10s preincubation*
control pDNA
oc form
sc form
GSH/selenite 300s preincubation**
GSH/selenite 45s preincubation**
GSH/selenite 10s preincubation**
GSH/selenite 300s preincubation*
GSH/selenite 45s preincubation*
GSH/selenite 10s preincubation*
control pDNA
oc form
sc form
GSH/selenite 300s preincubation**
selenite **
selenite*
GSH**
GSH*
selenite **
selenite*
control pDNA
oc form
sc form
GSH/selenite 10s preincubation**
GSH/selenite 300s preincubation*
GSH/selenite 10s preincubation*
control pDNA
