# Supplementary material for: Glutathione/Selenite Interaction Products as Potent Vascular Smooth Muscle Tone Modulators with Antihypertensive Effects in Spontaneously Hypertensive Rats
Source: Biol Trace Elem Res. 2026 Jun 24;204(9):7301–19. doi: 10.1007/s12011-026-05188-1 (PMC13437607; doi:10.1007/s12011-026-05188-1)
Supplement: Supplementary file 2 [file 12011_2026_5188_MOESM2_ESM.docx]

**Biological Trace Element Research**

**Supplementary Material**

**to**

**Glutathione/selenite interaction products as potent vascular smooth muscle tone modulators with antihypertensive effects in spontaneously hypertensive rats**

Lenka Tomasova^1^, Peter Balis^2^, Marian Grman^1^, Anna Zemancikova^2^, Sona Cacanyiova^2^, Miroslav Chovanec^3^, Karol Ondrias^1^, Anton Misak^1,^*

^1^ Institute of Clinical and Translational Research, Biomedical Research Center, Slovak Academy of Sciences, Dubravska cesta 9, 845 05 Bratislava, Slovak Republic; [marian.grman@savba.sk](mailto:marian.grman@savba.sk) (M.G.); [lenka.tomasova@savba.sk](mailto:lenka.tomasova@savba.sk) (L.T.); [karol.ondrias@savba.sk](mailto:karol.ondrias@savba.sk) (K.O.); [anton.misak@savba.sk](mailto:anton.misak@savba.sk) (A.M.)

^2^ Institute of Normal and Pathological Physiology, Centre of Experimental Medicine, Slovak Academy of Sciences, Sienkiewiczova 1,
813 71 Bratislava, Slovak Republic; [peter.balis@savba.sk](mailto:peter.balis@savba.sk) (P.B.); [anna.zemancikova@savba.sk](mailto:anna.zemancikova@savba.sk) (A.Z.); [sona.cacanyiova@savba.sk](mailto:sona.cacanyiova@savba.sk) (S.C.)

^3^ Cancer Research Institute, Biomedical Research Center, Slovak Academy of Sciences, Dubravska cesta 9, 845 05 Bratislava, Slovak Republic; [miroslav.chovanec@savba.sk](mailto:miroslav.chovanec@savba.sk) (M.C.)

* Correspondence: [anton.misak@savba.sk](mailto:anton.misak@savba.sk)

**Fig. S1** Snapshot series of the GSH/SeO_3_^2−^ mixture illustrating the color changes during incubation. Stock solutions of 200 mM GSH (in 100 mM sodium phosphate, 100 μM DTPA, pH 7.4) and 100 mM SeO_3_^2−^ (in 0.9% saline) were mixed at a 3:1 ratio to achieve the final concentrations of 150 and 25 mM, respectively. The mixture was incubated at 23±1°C and snapshots were taken at the indicated time points (in seconds). For *in vivo* experiments, the mixture incubated for 45 s was used

**Fig. S2** Representative time-dependent changes in APW-Ps obtained in anesthetized SHRs before (red) and after intravenous (i.v.) administration of GSH/SeO_3_^2−^ (150/25 mM) mixture incubated for 45 s at 23±1°C (blue). The bolus dose of GSH/SeO_3_^2−^ was standardized according to the body weight as 75/12.5 µmol/kg. The vertical dashed lines show the start of i.v. administration with a duration of 15 s. Definitions, units and abbreviations of APW-Ps derived from the APWs are detailed in our previous work [1]

**Fig. S3** Time-dependent changes in APW-Ps acquired in the anesthetized SHRs before (red) and after 3 sequential i.v. administrations of GSH/SeO_3_^2−^ (blue, green and black). Each bolus of the GSH/SeO_3_^2−^ mixture was administered at a dose of 75/12.5 µmol/kg

**Fig. S4** Time-dependent changes in APW-Ps obtained in 8 anesthetized SHRs before and after i.v. administration of the GSH/SeO_3_^2−^ mixture. The bolus dose of GSH/SeO_3_^2−^ was 75/12.5 µmol/kg. The vertical dashed lines show the start of the GSH/SeO_3_^2−^ administration with a duration of 15 s. Each color corresponds to the same rat

**Fig. S5** Time-dependent changes in APW-Ps obtained in 8 anesthetized SHRs before and after i.v. administration of SeO_3_^2−^ (12.5 µmol/kg). The vertical dashed lines show the start of the SeO_3_^2−^ administration with a duration of 15 s. Each color corresponds to the same rat

**Fig. S6** Time-dependent changes in APW-Ps obtained in 4 anesthetized SHRs before and after i.v. administration of the GSH (12.5 µmol/kg). The vertical dashed lines show the start of the GSH administration with a duration of 15 s. Each color corresponds to the same rat

**Fig. S7** Effect of saline and vehicle on APW-Ps. Time-dependent changes in APW-Ps were recorded in anesthetized SHRs before and after i.v. administration of saline **(A)** or vehicle **(B)**, and compared with the effect of GSH/SeO_3_^2−^ (75/12.5 µmol/kg) **(C)**. The area under the curve was analyzed over 180 s, with positive and negative components shown **(D)**. Positive components of SysBP, HR, DiaBP, PulseBP, AIr, and the negative components of dP/dt_max_ were evaluated and compared with the 180-s recordings of GSH and GSH/SeO_3_^2−^. Data are presented as individual values with means (±SD) and originate from Figs. S4 (GSH/SeO_3_^2−^, *n* = 8), S6 (GSH, *n* = 4), S7A (saline, *n* = 4) and S7B (vehicle, *n* =7). The vertical dashed lines show the start of the administration with duration of 15 s. Each color corresponds to the same rat

**Fig. S8** Time-dependent changes in APW-Ps acquired in the anesthetized SHRs before (red) and after 3 sequential i.v. administrations of GSH/SeO_3_^2−^ (blue, green and black). Each bolus of the GSH/SeO_3_^2−^ mixture was administered at a dose of 75/12.5 µmol/kg

**Fig. S9** Time-dependent changes in APW-Ps acquired in the anesthetized SHRs before (red) and after 2 sequential i.v. administrations of GSH/SeO_3_^2−^ (blue and green). Each bolus of the GSH/SeO_3_^2−^ mixture was administered at a dose of 75/12.5 µmol/kg

**Fig. S10** Effect of L-NAME and pentolinium preadministration on GSH/SeO_3_^2−^-induced changes in BP. After the i.v. administration of GSH/SeO_3_^2−^ (75/12.5 µmol/kg, not shown), the effects of L-NAME (25 mg/kg, dotted line,) and repeated administration of GSH/SeO_3_^2−^ (75/12.5 µmol/kg; black dotes) on SysBP and DiaBP were evaluated (A). The effects of GSH/SeO_3_^2−^ (75/12.5 µmol/kg) on BP after pretreatment with pentolinium (5 mg/kg) and L-NAME (25 mg/kg) (**B**). Each color corresponds to the same rat

**Fig. S11** Time-dependent changes in APW-Ps obtained in 7 anesthetized SHRs before and after i.v. administration of the LOS (12.5 µmol/kg). The vertical dashed lines show the start of the SeO_3_^2−^ administration with a duration of 15 s. Each color corresponds to the same rat

**Fig. S12** Time-dependent changes in APW-Ps obtained in 5 anesthetized SHRs before and after i.v. administration of the CAP (12.5 µmol/kg). The vertical dashed lines show the start of the SeO_3_^2−^ administration with a duration of 15 s. Each color corresponds to the same rat

**Fig. S13** The effects of LOS, CAP and GSH/SeO_3_^2−^ on APW-Ps. **(A)** After i.v. administration of LOS (12.5 µmol/kg), CAP (12.5 µmol/kg) or GSH/SeO_3_^2−^ (75/12.5 µmol/kg), the positive/negative components of the changes in APW-Ps were analyzed during the first 10 min. The total increase (orange-shaded bars)/decrease (blue-shaded bars) in APW-Ps was evaluated by calculating the area under the curve and expressed as a proportion of the APW-P basal area. The basal area was defined as the area described by the baseline over a 10-min interval. Only the relevant components (phases) were considered. **(B)** The changes in APW-Ps at the 20^th^ min were expressed as a percentage of the mean basal APW-P, which was defined as 100%. The basal APW-P (control) was calculated as the 30-s mean APW-P recorded prior to administration. The data are presented as individual values with means ± SD and originate from Figs. S4 (GSH/SeO_3_^2−^, *n* = 8), S11 (LOS, *n* = 7), and S12 (CAP, *n* = 5). The APW-P value for GSH/SeO_3_^2−^ obtained at 10^th^ min was considered as the steady-state level and was compared with the corresponding values of LOS and CAP at the 20^th^ min. Statistical analysis was performed using a one-way ANOVA followed by Tukey’s multiple comparison test with **P* < 0.05 (statistical results are provided in Tab. S3)

**Fig. S14** Vascular reactivity of mesenteric artery to H_2_O_2_. In contrast to KO_2_ (2 doses: 50 µM + 50 µM), the administration of H_2_O_2_ (2 doses: 50 µM + 100 µM) induced relaxation of mesenteric artery, which was diminished by CAT activity (> 110 U/mL)

**Fig. S15** Vascular reactivity of 3 types of SHR arteries to GSH+SeO_3_^2−^ application. Isolated segments of mesenteric artery (**A**), femoral artery (**B**) and thoracic aorta (**C**) were preconstricted with NOR (1 μM), Ser (1 μM) and NOR (10 μM), respectively. Vascular tension in aortic rings without NOR preconstriction was normalized to constriction induced by 125 mM KPPS (**D**). In all other cases (A-C), responses were normalized to the NOR/Ser preconstriction level. Once a steady-state level was reached, GSH (0.5 mM) and subsequently SeO_3_^2−^ (0.04 mM) were applied into the organ bath in the presence of SOD and CAT (both at activity > 110 U/mL; blue traces) or in their absence (red traces). The baseline represents ~ 100% and serves as the control

**Fig. S16** The vasoactive effects of GSH+SeO_3_^2−^ on mesenteric arteries isolated from Wistar rats. Mesenteric artery rings were either denuded of endothelium (ED) or co-applied with SOD (activity > 150 U/mL) and subsequently subjected to GSH (0.5 mM) and SeO_3_^2−^ (0.04 mM). Traces represent the mean±SD (except ED) changes in vascular tension, with the number of experiments indicated in parentheses. Vascular tension was normalized to the NOR-preconstricted (1 µM) plateau (set as 100%)

**Fig. S17** Vasoactive effects of GSH+SeO_3_^2−^ in arteries after pharmacological modulation with L-NAME, glibenclamide, and TEA. Isolated rings of mesenteric artery and thoracic aorta were pretreated for 20 min with L-NAME (300 µM), glibenclamide (40 µM), or TEA (1 mM), and subsequently preconstricted with NOR. GSH (0.5 mM) and SeO_3_^2−^ (0.04 mM) were then applied to the organ bath either in the absence (red symbols and lines) or presence (blue symbols and lines) of SOD and CAT (activity > 110 U/mL). The upper panel represents vascular tension measured at 12 min after GSH+SeO_3_^2−^ application. These values were extracted from individual recordings (dashed lines) presented in the lower panels. Solid lines with the grey-shaded area represent the control 95% confidence interval (CI), corresponding to GSH+SeO_3_^2−^ responses without pharmacological pretreatment. Vascular tension was normalized to the NOR-preconstricted plateau (set as 100%)

**Fig. S18** The effect of SOD and CAT on the spectrophotometric properties of the GSH/SeO_3_^2−^ (0.5/0.04 mM) mixture. Time-resolved UV-VIS spectra of GSH/SeO_3_^2−^ were recorded in 100 mM sodium phosphate buffer (supplemented with 0.1 mM DTPA, pH 7.4) at 37±1 °C every 20 s for 10 min, either in the absence presence of CAT (activity > 100 U/mL), SOD (activity > 100 U/mL), or their combination. A 35 µL aliquot of GSH (10 mM stock solution prepared in ddH2O) was added to 665µL of sodium phosphate buffer containing 0.04 mM SeO_3_^2−^ (corresponding to 20 µL of a 1.4 mM stock solution prepared in ddH2O). The final reaction mixtures additionally contained SOD (25 µL; 3200 U/mL in 50 mM sodium phosphate) and/or CAT (10 µL; 7000 U/mL in 50 mM sodium phosphate), as specified. The last panel compares spectra recorded at 0 min and 10 min. For samples containing CAT, the background spectrum of CAT alone was subtracted

**Fig. S19** Snapshot series illustrating the effect of ROS on the color changes in the GSH/ SeO_3_^2−^ mixture. Stock solutions of 10 mM GSH (in 100 mM sodium phosphate, 100 μM DTPA, pH 7.4) and 0.8 mM SeO_3_^2−^ (in 100 mM sodium phosphate, 100 μM DTPA, pH 7.4) were mixed at a 1:1 ratio to attain the final concentrations of 5 and 0.4 mM, respectively (upper row). SOD and CAT were added to the GSH solution prior to SeO_3_^2−^ addition (final activity 100 U/mL, lower row). The mixtures were incubated at 23±1°C and snapshots were taken at the indicated time points (in seconds)

**Fig. S20** Spectrophotometric and reducing properties of GSH/SeO_3_^2−^. (**A**) UV-Vis spectrum of 25 mM SeO_3_^2−^ (black) and 150 mM GSH (dashed black). Time-resolved UV-VIS spectra of GSH/SeO_3_^2−^ (150/25 mM) incubated at 24±1 °C for 20 (red), 30 (red long dash), 40 (red medium dash), 60 (red short dash), 80 (red dotted), 90 (blue), 120 (blue long dash), 150 (blue medium dash), 180 (blue short dash), 210 (blue dotted), 240 (green), 270 (green long dash), 300 (green medium dash) and 330 s (green short dash). A 333 µL aliquot of SeO_3_^2−^ (100 mM in 0.9% saline) was added to 1000 µL of GSH (200 mM; prepared in 100 mM sodium phosphate buffer supplemented with 100 μM DTPA, pH 7.4) and incubated for the specified time. All UV-VIS spectra were recorded every 20 or 30 s at 24±1 C. (**B**) Time-dependent reduction of the ^•^cPTIO radical (100 µM) by the GSH/SeO_3_^2–^ (150/25 mM) mixture incubated at 24±1 °C for the following times: 5 (red), 30 (green) or 45 s (cyan), and for 1 (pink), 3 (dark yellow), 5 (blue) or 30 min (black). After incubation, the mixtures were diluted 1000-fold to the concentration of 150/25 µM before addition to ^•^cPTIO. **(C)** Effect of incubation time on ABS at 560 nm, compared at 20 min

| **APW-P** | **Interaction** | **Time as a factor** | **Treatment as a factor** |
| --- | --- | --- | --- |
| **SysBP** | F (1, 14) = 2.429 and P = 0.1414 | F (1, 14) = 70.63 and P < 0.0001 | F (1, 14) = 0.1818 and P = 0.6763 |
| **DiaBP** | F (1, 14) = 3.123 and P = 0.0990 | F (1, 14) = 104.9 and P < 0.0001 | F (1, 14) = 0.7249 and P = 0.4089 |
| **PulBP** | F (1, 14) = 1.273 and P = 0.2781 | F (1, 14) = 25.66 and P = 0.0002 | F (1, 14) = 0.2581 and P = 0.6193 |
| **AI_r_** | F (1, 14) = 2.156 and P = 0.1642 | F (1, 14) = 51.11 and P < 0.0001 | F (1, 14) = 2.173 and P = 0.1626 |
| **HR** | F (1, 14) = 0.3012 and P = 0.5918 | F (1, 14) = 42.06 and P < 0.0001 | F (1, 14) = 0.9349 and P = 0.3500 |
| **dP/dt_max_** | F (1, 14) = 7.479 and P = 0.0161 | F (1, 14) = 53.29 and P < 0.0001 | F (1, 14) = 7.237 and P = 0.0176 |
|  |  |  |  |
| Time = (0, 10 min) RMQ: 0 min = control APW-P | | | |
| Treatment = (SeO_3_^2−^, mixture) | |  |  |

**Tab. S1** Statistical results of two-way ANOVA comparing SeO_3_^2−^ and GSH/SeO_3_^2−^ treatments in SHRs at two different time points. The analysis includes the factors Time and Treatment, with a significance threshold set at *P* < 0.05. The data corresponding to this analysis are graphically represented in **Fig. 5**

| **Artery type** | **Phase** |
| --- | --- |
| **Mesenteric** | F (1.013, 8.101) = 43.93 and P = 0.0002 |
| **Femoral** | F (1.207, 6.034) = 16.94 and P = 0.0052 |
| Phases for mesenteric a. = (control, transient increase, decrease at 12^th^ min)  Phases for femoral a. = (control, transient decrease, transient increase, decrease at 12^th^ min) | |

**Tab. S2** Statistical results of one-way ANOVA with repeated measurements comparing the effect of GSH+SeO_3_^2−^ on the control vascular tone at different phases (factor) of vasoactivity. A significance threshold is considered *P* < 0.05. The data corresponding to this analysis are graphically represented in **Fig. 8**

| **APW-P** | **Treatment** | |  |  |  |  |  |  |
| --- | --- | --- | --- | --- | --- | --- | --- | --- |
| **SysBP** | F (2, 17) = 1.099 and P = 0.3556 |  | |  | |  | |  |
| **DiaBP** | F (2, 17) = 1.048 and P = 0.3723 | |  | |  | |  |  |
| **PulBP** | F (2, 17) = 1.137 and P = 0.3439 | |  | |  | |  |  |
| **AI_r_** | F (2, 17) = 1.278 and P = 0.3041 | |  | |  | |  |  |
| **HR** | F (2, 17) = 6.598 and P = 0.0076 | |  | |  | |  |  |
| **dP/dt_max_** | F (2, 17) = 2.642 and P = 0.1002 | |  | |  | |  |  |
| Treatment = (LOS, CAP, mixture) | | |  |  |  |  |  |  |

**Tab. S3** Statistical results of one-way ANOVA comparing the steady-state APW-Ps between LOS-, CAP- and GSH/SeO_3_^2−^-treated (factor) SHRs. A significance threshold is considered *P* < 0.05. The data corresponding to this analysis are graphically represented in **Fig. S13B**

**References:**

1. Kurakova L, Misak A, Tomasova L, Cacanyiova S, Berenyiova A, Ondriasova E, et al. Mathematical relationships of patterns of 35 rat haemodynamic parameters for conditions of hypertension resulting from decreased nitric oxide bioavailability. Exp Physiol. 2020;105(2):312-34. doi: 10.1113/ep088148.
